# A Novel Prognostic Ferroptosis-Related lncRNA Signature Associated with Immune Landscape in Invasive Breast Cancer

**DOI:** 10.1155/2022/9168556

**Published:** 2022-03-20

**Authors:** Shuang Shen, Danhe Yang, Yumin Yang, Yanqi Chen, Jing Xiong, Xiaochi Hu

**Affiliations:** ^1^Department of Breast & Thyroid Surgery, Third Affiliated Hospital of Zunyi Medical University/First People's Hospital of Zunyi, Zunyi, China; ^2^Department of Gynecology, Affiliated Hospital of Zunyi Medical University, Zunyi, China

## Abstract

Breast cancer (BC) represents the most common form of malignant tumors in women. However, the effectiveness of BC immunotherapy remains very low. Ferroptosis is a recently described form of programmed cell death which has unique characteristics, and associated long-chain noncoding RNAs (lncRNA) are thought to influence the occurrence and development of a variety of tumors. We identified 1,636 lncRNAs associated with ferroptosis in BC patients. 299 differentially expressed ferroptosis-related lncRNAs were subjected to univariate, LASSO regression, and multivariate Cox regression analyses to construct a ten ferroptosis-related lncRNA signature. This ten ferroptosis-related lncRNA signature performed very well in predicting survival of BC patients, and the risk score of the mRNA signature was identified as an independent prognostic factor in this cancer entity. In addition, the signature could be used to predict the immune landscape of BC patients. Low-risk patients had enriched immune-related pathways and more infiltration of most types of immune cells. The signature was also associated with the tumor mutation burden in BC. The results have allowed us to assess the potential for immunotherapy targets exposed by this model. The ferroptosis-related lncRNA risk model reported in the current study has clinical utility in BC prognosis and predicted immunotherapy response.

## 1. Introduction

The highest incidence of malignant tumors in women is due to breast cancer (BC), and this is also the cancer responsible for the largest number of female deaths [1]. Data released by the International Agency for Research on Cancer (IARC) recorded 2.26 million new cases of BC in 2020, an incidence which replaces lung cancer as the leading form of cancer throughout the world [2]. Early BC diagnosis in combination with improved treatment options, such as surgery, chemotherapy, endocrine therapy, and targeted therapy, has reduced the mortality rate by 30% compared with 1990 [3]. However, conventional treatment regimens remain largely ineffective for patients with advanced or metastatic forms of the disease, and most of these patients will still die from their tumors. Clearly, deficiencies remain in terms of clinicopathological indicators, and there is a pressing need to improve prognostic techniques.

Ferroptosis represents a form of programmed cell death which depends on iron-mediated oxidative damage of cell membranes [4, 5]. Recent studies have demonstrated the involvement of ferroptosis regulation in the occurrence and response to treatment of various tumor types [6–8]. Many proteins have been identified, such as HRAS, NRAS, KRAS, TP53, NFE2L2, and HIF, which affect proliferation and migration of tumor cells and are associated with ferroptosis [9]. Meanwhile, as a subset of RNA molecules, long noncoding RNA (lncRNA) regulates gene expression [10, 11]. The expression of such proteins may be subject to regulation by lncRNA, identification of which would improve our understanding of tumor progression. Tang et al. [12] indicated the utility of ferroptosis-related lncRNA for the prognosis of head and neck squamous cell carcinoma. Moreover, Zheng et al. [13] described the regulatory effects of ferroptosis-related lncRNA on the glioma microenvironment and its relationship to radiotherapy response and immunity. However, specific roles for ferroptosis-related lncRNA are still unclear. Few studies have taken a systematic approach to relating lncRNA sequence information to immunotherapy responses and BC patient survival.

During the current study, we used BC gene expression profile data from The Cancer Genome Atlas (TCGA) to construct a novel prognostic risk signature based on ten differentially expressed ferroptosis-related lncRNAs. We explored the relationship between the model and immunotherapy response and tumor mutation burden (TMB). Our findings have allowed us to construct a nomogram to predict the overall survival (OS) of BC patients.

## 2. Results

### 2.1. Identification of Differentially Expressed Ferroptosis-Related lncRNA

Ferroptosis-related genes (Table S1) were identified from FerDb, and an expression matrix of 246 genes and 14,086 lncRNAs was extracted from the TCGA-BRCA set. In order to identify differentially expressed lncRNA, 1,636 ferroptosis-related lncRNAs were screened and expression compared by Pearson's correlation (∣*R*^2^ | >0.4 and *p* < 0.001). The analysis revealed that 299 ferroptosis-related lncRNAs were differentially expressed (196 upregulated and 103 downregulated) (Table S2). The experimental scheme is illustrated by the flow diagram shown in Figure S1.

### 2.2. Construction and Verification of BC Risk Model Based on Ferroptosis-Related lncRNA

**Figure 1:**
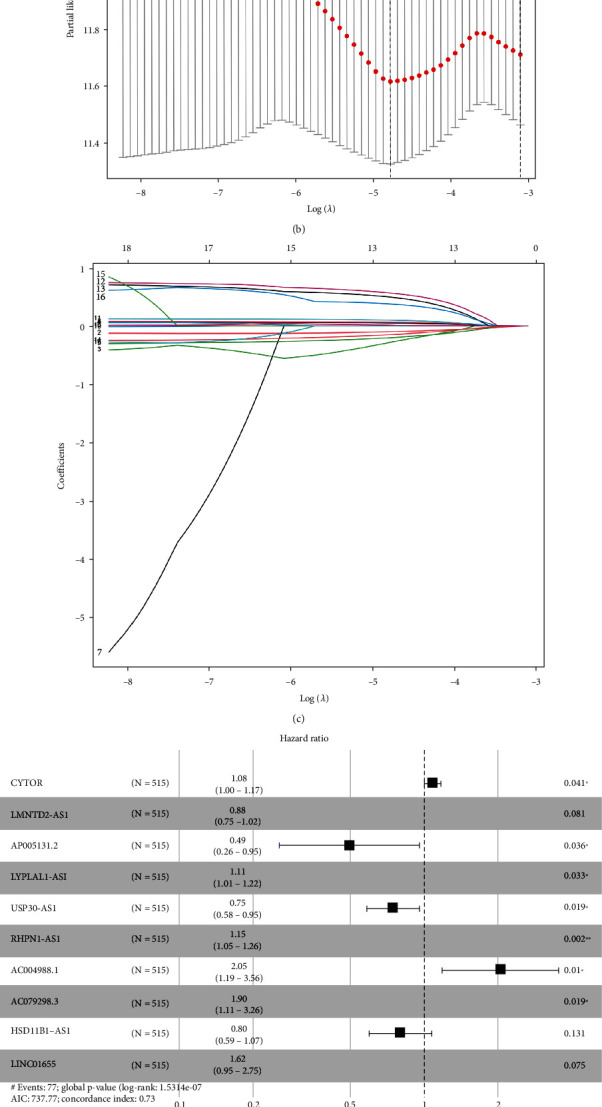
Risk model for BC patients based on ferroptosis-related lncRNAs. (a) Univariate Cox regression analysis showed that the included ferroptosis-related lncRNAs were significantly associated with clinical survival prognosis. (b) The tuning parameters (log l) of OS-related proteins were selected to cross-verify the error curve. According to the minimal criterion and 1-se criterion, perpendicular imaginary lines were drawn at the optimal value. (c) The LASSO coefficient profile of 13 OS-related lncRNAs and perpendicular imaginary line were drawn at the value chosen by 10-fold cross-validation. (d) Ten ferroptosis-related lncRNAs with independent prognosis and survival correlation were shown by multivariate Cox regression analysis. (e) Sankey relational diagram for the ferroptosis-related genes and the lncRNAs used to construct the risk model.

**Figure 2:**
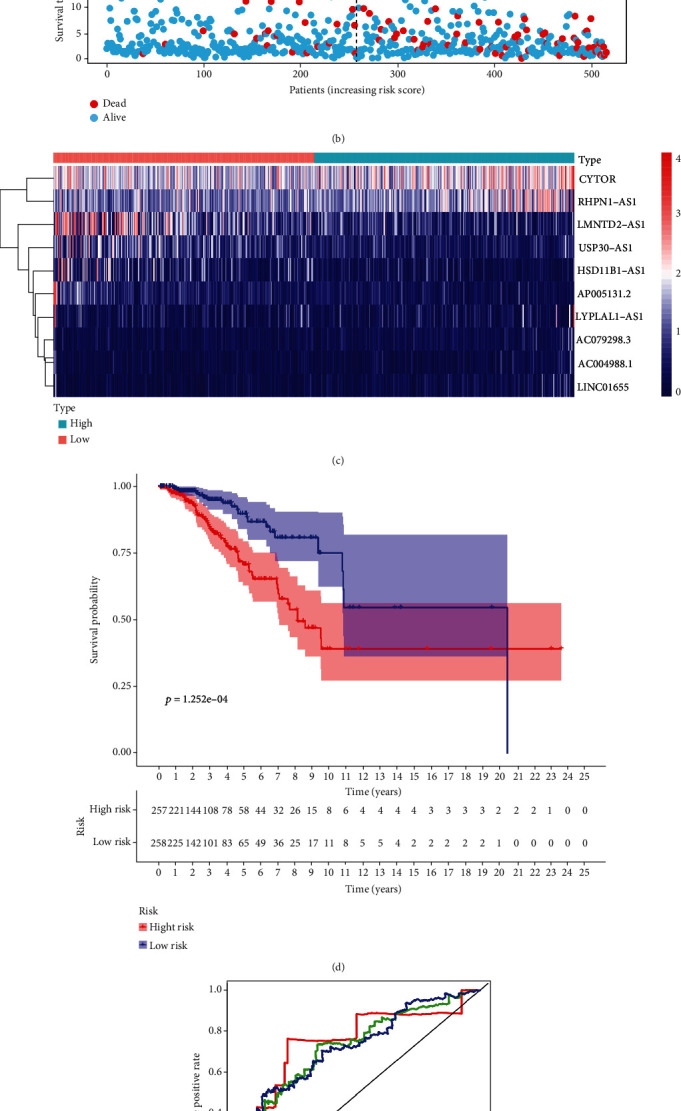
Prognostic value of the risk patterns of the 10 ferroptosis-related lncRNAs in the TCGA-BRCA training set. (a) Distribution of ferroptosis-related lncRNA model-based risk score. (b) Different patterns of survival status and survival time between the high- and low-risk groups. (c) Clustering analysis heat map shows the expression standards of the 10 prognostic lncRNAs for each patient. (d) Kaplan-Meier survival curves of the OS of patients in the high- and low-risk groups. (e) The AUC values of the patients' 1-, 3-, and 5-year survival rate.

Nineteen differentially expressed ferroptosis-related lncRNAs associated with prognosis were identified from the TCGA-BRCA training set by univariate Cox regression analysis (Figure 1(a)). Use of LASSO Cox regression to prevent overfitting of the model revealed 13 ferroptosis-related lncRNAs for subsequent multivariate analysis (Figures 1(b) and 1(c)). Of these, 10 ferroptosis-related lncRNAs related to BC OS emerged from multivariate Cox regression (Figure 1(d)) (Table S3). The 10 ferroptosis-related lncRNAs constitute a prognosis independently related to OS in the training cohort and were used to build a risk model. The coexpression relationship between the ferroptosis-related genes and the lncRNAs used to construct the model is shown in the Sankey diagram (Figure 1(e)). Using the median score of the risk signature, patients were divided into two subgroups, high and low risk (Figure 2(a)). Survival status and survival times of patients in the two risk groups are shown in Figure 2(b) with relative expression standards of lncRNAs for each patient in Figure 2(c). Kaplan-Meier analyses revealed that high-risk patients showed poorer OS (*p* < 0.001) (Figure 2(d)). The area under the ROC curve (AUC) of the 1-year, 3-year, and 5-year survival of BC patients was assessed to be 0.755, 0.731, and 0.721 (Figure 2(e)). It demonstrated that the model has excellent precision to predict survival.

**Figure 3:**
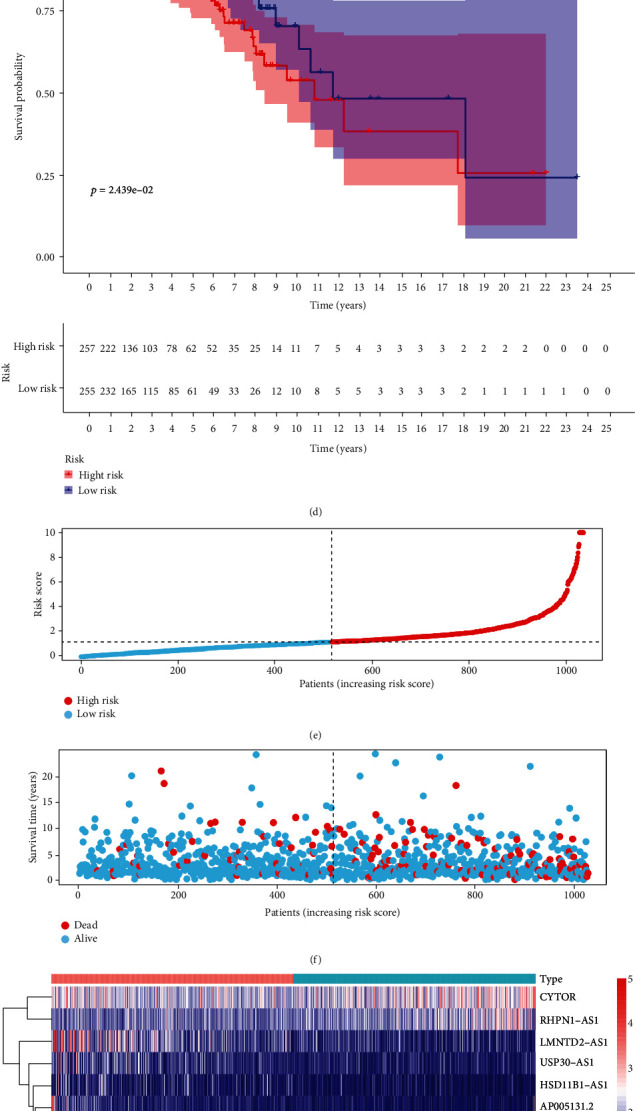
Prognostic value of the risk model of the 10 ferroptosis-related lncRNAs in the TCGA-BRCA testing and entire sets. (a) Distribution of ferroptosis-related lncRNA model-based risk score for the testing set. (b) Patterns of the survival time and survival status between the high- and low-risk groups for the testing set. (c) Clustering analysis heat map shows the display levels of the 10 prognostic lncRNAs for each patient in the testing set. (d) Kaplan-Meier survival curves of the OS of patients in the high- and low-risk groups for the testing set. (e) Distribution of the ferroptosis-related lncRNA model-based risk score for the entire set. (f) Patterns of the survival time and survival status between the high- and low-risk groups for the entire set. (g) Clustering analysis heat map shows the expression levels of the 10 prognostic lncRNAs for each patient for the entire set. (h) Kaplan-Meier survival curves of OS of patients in the low- and high-risk groups for the entire set.

A unified formula was used to calculate the risk score for each patient in the test set and in the entire cohort. Risk distributions, survival status, and survival time together with expression of ferroptosis-related lncRNAs in the TCGA-BRCA cohort and test set are shown in Figure 3. OS among members of the low-risk group was higher than that of the high-risk group for both the test set and the entire cohort (Figures 3(d) and 3(h)). We conclude that expression levels of ferroptosis-related lncRNA can be correlated with prognosis for BC patients.

**Figure 4:**
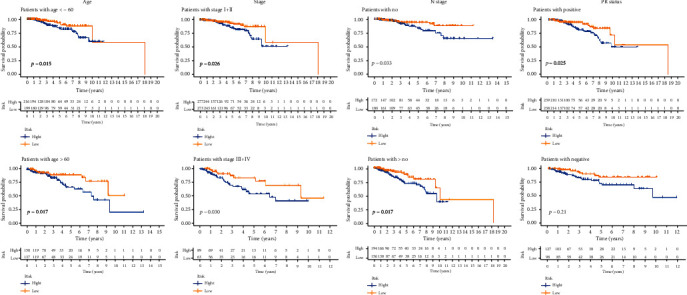
Kaplan-Meier curves of OS differences stratified by age, TNM stage, N stage, and progesterone receptor (PR) status between the high- and low-risk groups in the TCGA-BRCA entire set.

Differences in OS, together with clinicopathological characteristics, were analyzed according to the low-risk and high-risk groups in the entire TCGA-BRCA cohort. Patients were assigned to subgroups according to age, TNM stage, N stage, and progesterone receptor (PR) status. The OS of the low-risk group was found to be consistently better than that of the high-risk group (Figure 4).

### 2.3. Verification of the Model by PCA

A PCA was performed for the total gene expression profile, 246 ferroptosis-related genes, 1,636 ferroptosis-related lncRNAs, and risk signature classified by expression profiles of the 10 ferroptosis-related lncRNAs to assess differences between the low-risk and high-risk groups (Figures S2A-S2C). The results show different distributions for the low-risk and high-risk groups (Figure S2D), indicating that the prognostic model distinguishes between the two groups.

### 2.4. Distinction between Groups by Gene Set Enrichment Analysis

Gene set enrichment analysis (GSEA) revealed that immune and tumor-related pathways, such as primary immunodeficiency, T cell receptor signaling pathway, intestinal immune network for IgA production, hematopoietic cell lineage, type I diabetes mellitus, allograft rejection, cytokine-cytokine receptor interaction, antigen processing and presentation, graft-versus-host disease, chemokine signaling pathway, and natural killer cell-mediated cytotoxicity, showed appropriate hallmarks of regulation in the low-risk group (Figure S3 and Table S4).

### 2.5. Correlation of Tumor-Infiltrating Immune Cells, Immunosuppressive Molecules, and Immunotherapy Score with Risk Assessment Model

**Figure 5:**
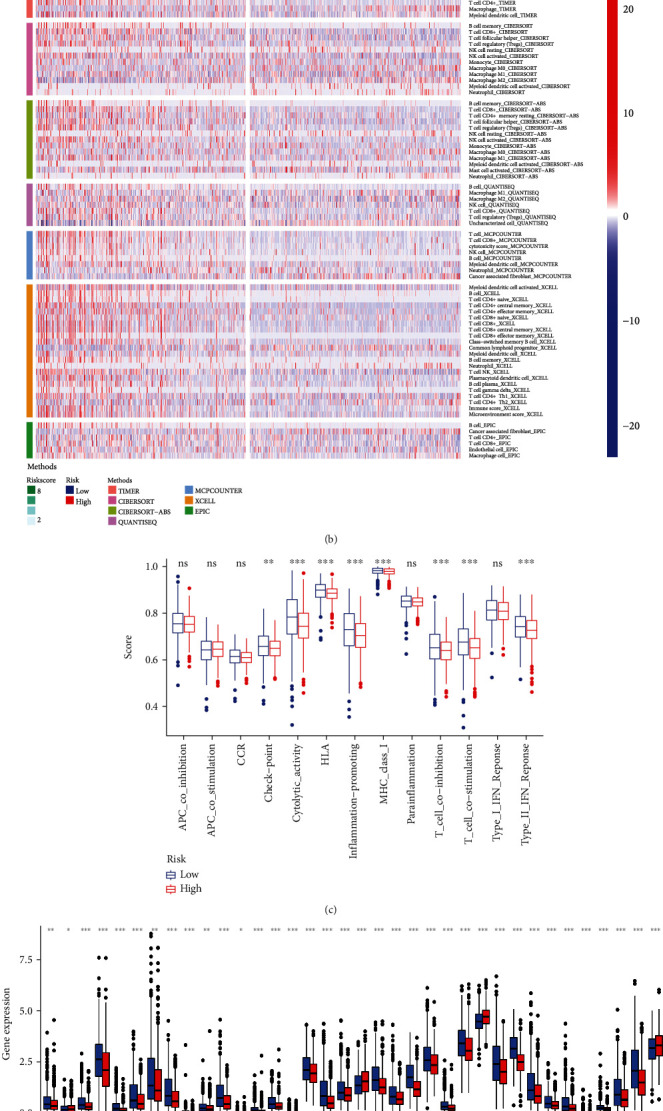
Estimation of the tumor-infiltrating immune cells, immunosuppressive molecules, and cancer immunotherapy response using the ferroptosis-related lncRNA model in the TCGA-BRCA entire set. (a) Patients in the low-risk group were more positively associated with tumor-infiltrating immune cells such as B cells, CD8+ T cells, and CD4+ T cells as shown by Spearman correlation analysis. (b) The heat map of immune responses among the high-risk and low-risk groups based on ferroptosis-related lncRNA signatures. (c) ssGSEA for the association between immune cell subpopulations and related functions. (d) Expression of immune checkpoints among the high and low BC risk groups. (e–h) IPS comparison between the low-risk groups and the high-risk groups in the TCGA-BRCA entire set in the CTLA4 negative/positive or PD-1 negative/positive groups. CTLA4_positive or PD1_positive represented anti-CTLA4 or anti-PD-1/PD-L1 therapy, respectively (all *p* < 0.001).

Expression levels and activities of immune cells, pathways, or functions were assessed by GSEA and correlated with our lncRNA risk model in the 1,027 TCGA-BRCA samples. The results reveal differences in the immune index between the low-risk and high-risk groups with immune cells, such as CD4 and CD8 T cells, showing greater infiltration in the former group (Figures 5(a) and 5(b)). Immune pathways, such as checkpoint, cytolytic activity, HLA, inflammation, MHC, T cell functions, and IFN response, all show lower activity levels in the high-risk group, compared with the low-risk group (*p* < 0.01) (Figure 5(c)). The correlation between the risk group and immunotherapy biomarkers, such as CTLA4, PDCD1, CD274, and LAG3, revealed higher levels of expression in the low-risk group (*p* < 0.001) (Figure 5(d)). The low-risk group showed higher immunophenoscores (*p* < 0.001) for the four categories of immunotherapy, indicating the utility of lncRNA classification as a predictor of immunotherapy efficacy (Figures 5(e)–5(h)).

### 2.6. Correlation of TMB with Risk Model

**Figure 6:**
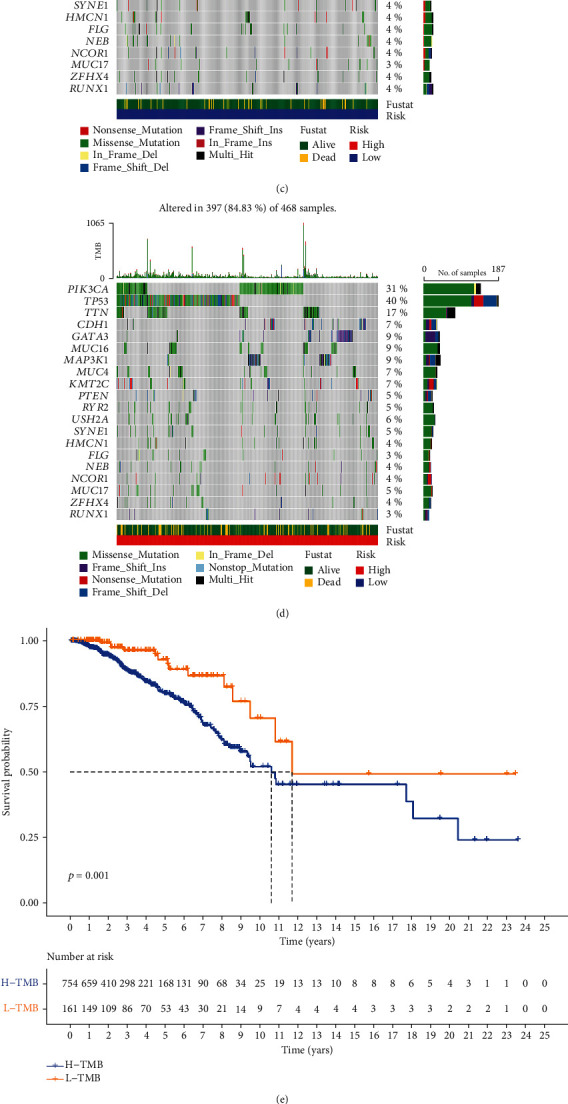
Correlation of tumor mutation burden with the ferroptosis-related lncRNA risk model. (a) Differences in TMB scores between the high-risk and low-risk groups (*p* < 0.001). (b) Association between TMB score and their distribution in the low- and the high-risk groups. (c, d) Waterfall plot displays mutation information of the genes with high mutation frequencies in the low-risk group (c) and the high-risk group (d). (e) Kaplan-Meier curve analysis of OS is shown for patients classified according to the TMB score. (f) Kaplan-Meier curve analysis of OS is shown for patients classified according to the TMB score and ferroptosis-related lncRNA model.

Recent studies have demonstrated the relationship between TMB and OS for multiple cancer types after immunotherapy [14, 15]. The mutation data of the TGCA set was analyzed using the R package *maftools*.  Figure 6(a) shows the TMB score in the high-risk group exceeded that in the low-risk group (*p* < 0.001).  Figure 6(b) shows a significant positive correlation between the model risk grouping and the TMB (*p* < 0.001, *R* = 0.15). The top 20 driver genes with the highest frequency of change between the high-risk and low-risk groups are shown in Figures 6(c) and 6(d). TMB scores for each group were calculated using TGCA-BRCA somatic mutation data, revealing significant differences between the two groups. Patients in the high-risk group had higher TMB scores. Using the best cutoff value according to the mutation effect predictor, the total samples were divided into two groups according to TMB. Survival times for patients in the low TMB group were significantly higher than that for the high TMB group (*p* < 0.001) (Figure 6(e)). Combining survival analysis for the risk model and TMB groups revealed that the low-risk group with low TMB had the highest survival rate, while the high-risk group with high TMB had the worst survival rate (*p* < 0.001) (Figure 6(f)). We conclude that the ferroptosis-related lncRNA risk model may have a greater prognostic value than TMB status alone.

### 2.7. Identification of Novel Candidate Compounds Targeting Ferroptosis-Related lncRNA Models

In order to discover the potency of the ferroptosis-related risk model as a biomarker for predicting the response of BC patients to drugs (including chemotherapy and targeted therapies), we inferred the IC50 values of 137 drugs in TCGA-BRCA patients. We found that patients in the low-risk group may be more sensitive to olaparib (AZD.2281), veliparib (ABT-888), gefitinib, metformin, methotrexate, etc., while patients in the high-risk group may be more sensitive to erlotinib, lapatinib, imatinib, pazopanib, etc. (Figure S4).

### 2.8. Comparison of Risk Model with Clinical Characteristics of Breast Cancer

**Figure 7:**
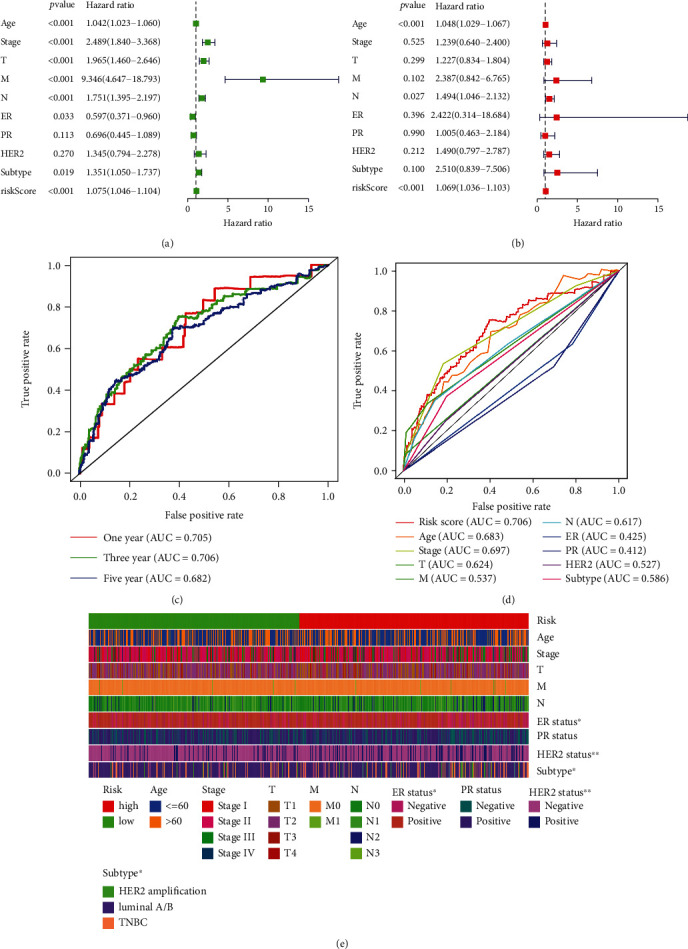
Assessment of the prognostic risk model of the ferroptosis-related lncRNAs and clinical features in BC in the TCGA-BRCA entire set. (a, b) Univariate (a) and multivariate (b) analyses of the clinical characteristics and risk score with the OS. (c) Concordance indexes of the risk score and clinical characteristics. (d) ROC curves of the clinical characteristics and risk score. (e) Heat map for ferroptosis-related lncRNA prognostic signature and clinicopathological manifestations (^∗∗∗^<0.001, ^∗∗∗^<0.01, and ^∗^<0.05).

Univariate and multivariate Cox regression analyses were used to analyze established clinicopathological BC characteristics in the context of our risk model. Single-factor regression analysis shows that the HR of the risk score was 1.075 with a 95% confidence interval (CI) of 1.046−1.104 (*p* < 0.001) (Figure 7(a)). Multivariate regression analysis produced a HR of the risk score and 95% confidence interval (CI) of 1.069 and 1.036−1.103 (*p* < 0.001) (Figure 7(b)), respectively. The above results indicate that our risk model has no correlation with established clinicopathological parameters, such as age, tumor grade, TNM staging system, or molecular BC classification, and is an appropriate independent prognostic tool for BC patients. An additional evaluation was performed to compare the prognostic utility of our model with established clinicopathological indicators by means of the ROC curve (Figure 7(c)). Our risk model produced a larger area under the curve than other clinicopathological indicators (Figures 7(d) and 7(e)). We conclude that our model, based on 10 ferroptosis-related lncRNAs, has advantages over established clinicopathological characteristics, such as tumor grade, TNM staging system, and molecular BC classification and also shows robust reliability.

### 2.9. Construction and Evaluation of Risk Model Nomogram

**Figure 8:**
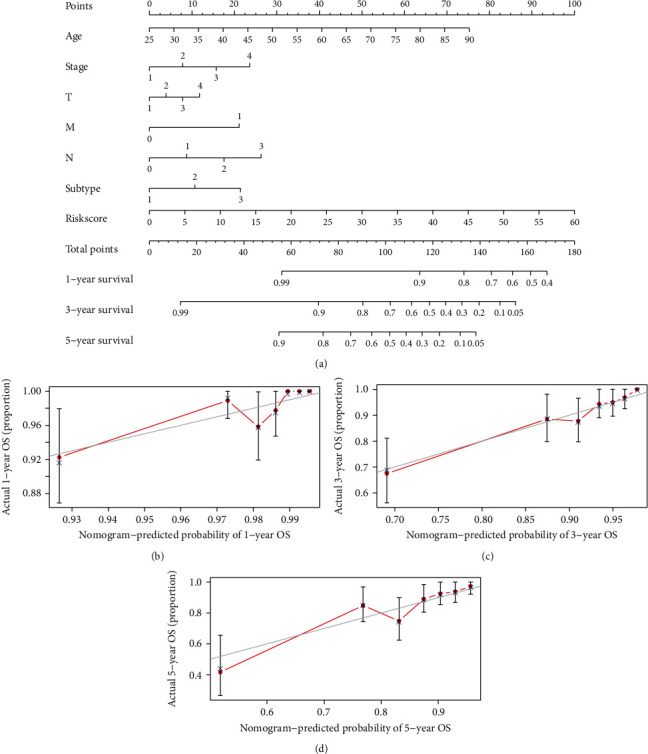
Construction and evaluation of a prognostic nomogram. (a) The nomogram predicts the probability of the 1-, 3-, and 5-year OS. (b–d) The calibration plot of the nomogram predicts the probability of the 1-, 3-, and 5-year OS.

A nomogram with integrated prognostic tools, including risk model and clinicopathological characteristics, was constructed in order to predict the 1-, 3-, and 5-year OS of BC patients (Figure 8(a)). The relevant calibration curve shows excellent agreement between OS at 1, 3, and 5 years and predicted OS rates (Figures 8(b)–8(d)).

### 2.10. Validation of the Expression Levels of Ten of the Ferroptosis-Related lncRNAs in Breast Cancer Cell Lines and Normal Human Breast Epithelial Cell Line

For validating the expression levels of the ferroptosis-related prognostic lncRNAs, we detected ten ferroptosis-related prognostic lncRNA expression levels in breast cancer cell lines (MCF7, SKBR3, and MDA-MB-231) and a normal human breast epithelial cell line (MCF10A) by using the RT-qPCR assay. Our results showed that CYTOR, LMNTD2-AS1, LYPLAL1-AS1, USP30-AS1, RHPN1, LINC01655, AP005131.2, AC004988.1, and AC079289.3 were upregulated in breast cancer cell lines (SKBR3) compared with the normal human breast epithelial cell line (MCF10A), and HSD11B1-AS1 was downregulated in breast cancer cell lines (MCF7, SKBR3, and MDA-MB-231) (Figure S5).

## 3. Discussion

Malignant BC tumors pose one of the greatest threats to women's health with resistance to chemotherapy drugs being an increasing problem [16]. Many chemotherapeutic drugs act by inducing tumor cell death by activation of apoptosis pathways [17]. If the tumor cells evade apoptosis, then resistance to chemotherapy is a potential consequence. Thus, strategies to overcome chemotherapy resistance are urgently required. The more recently discovered ferroptosis pathway of programmed cell death has some characteristics, which distinguishes it from better-known apoptosis pathways. The use of ferroptosis inducers has shown great potential in activating programmed cell death in tumor cells and also in enhancing sensitivity to well-established tumor cytotoxic drugs [18]. For example, the chemotherapeutic drug, cisplatin, shows significantly increased antitumor activity when administered in combination with a ferroptosis inducer [19]. Such studies demonstrate the suitability of the ferroptosis pathway as a target for anticancer drugs and herald a new era in clinical tumor treatment [18, 20].

lncRNAs are more than 200 nucleotides in length and lack an open reading frame [21]. They are known to have signaling roles, including regulation of the tumor cell ferroptosis pathway in which a dual role has been suggested. Some studies have linked inhibition of tumor cell ferroptosis with the promotion of cancer growth. For example, upregulation of the lncRNA, LINC00336, in lung cancer cells has been shown to activate the LSH/ELAVL1/LINC00336 axis, resulting in inhibition of ferroptosis and increased tumor formation [22]. By contrast, the findings of Mao et al. [23] demonstrated that the lncRNA, P53RRA, has an impact on the regulation of the p53 pathway, resulting in cell cycle arrest, apoptosis, and ferroptosis. Thus, P53RRA acts as a tumor suppressor. Thus, the balance between stimulatory and inhibitory effects of lncRNA on ferroptosis may have great significance for whole-body homeostasis. To date, few studies have investigated the correlation between ferroptosis-related lncRNA and BC. The current study is aimed at extending such knowledge by constructing a prognostic model based on ferroptosis-related lncRNA.

The current study used differentially expressed ferroptosis-related lncRNAs identified in the TCGA-BRCA cohort. Prognosis-related lncRNAs were selected and used to construct a novel risk signature to predict the OS of BC patients. Among the lncRNAs investigated, CYTOR was upregulated in colorectal cancer samples and associated with poor prognosis, having a role in proliferation and metastasis [24]. Moreover, *in vivo* and *in vitro* experiments have associated a longer OS in cervical cancer patients with low expression of the lncRNA, USP30-AS1 [25]. The current study reveals the involvement of further novel lncRNAs in BC. In our cell line experiment, these two lncRNAs were also detected to be significantly upregulated in breast cancer cell lines, which may be closely linked to tumor formation, and specific biological behaviour still needs additional research and verification. We divided BC patients into the high- and low-risk groups, depending on the median value of the risk model. Clinical outcomes were consistently better for the low-risk group. Multivariate Cox regression analysis demonstrated that the ferroptosis-related lncRNA model is an independent prognosis of OS. ROC analysis demonstrated the superiority of our risk model in predicting OS of BC patients by comparison with established clinicopathological characteristics. We extended our model to build a hybrid nomogram predicting 1-year, 3-year, and 5-year OS. The calibration curve shows excellent agreement between model predictions and ensuing OS. We conclude that our risk model, based on ten ferroptosis-related lncRNAs, has a high degree of accuracy and may contribute to the search for new biomarkers.

Recent findings on immune checkpoints have opened a new era in the treatment of malignant tumors [26, 27]. The suggestion has been made that expression of TILs has predictive value for immunotherapy response and prognosis of melanoma, BC, and colorectal cancer [28]. Functional enrichment analysis performed during the current study showed a higher degree of enrichment for immune-related pathways for the low-risk group and that CD4+ and CD8+ T cells are more abundant in tumor infiltration. Together with immune function analysis, these results indicate a better immune function for the low-risk group compared with the high-risk group. Clinical studies of neoadjuvant therapy have found that higher expression of PD-L1 mRNA or protein constitutes an independent positive predictor of pathological response [29, 30]. PD-L1 expression is also used by researchers as an indicator of BC prognosis and predicted survival. However, many controversies remain. In a study of 870 BC patients, Qin et al. [31] associated disease-free survival (DFS), metastasis-free survival (MFS), and OS negatively with high PD-L1 expression, suggesting that high PD-L1 expression is an indicator of poor prognosis in BC patients. The current study reveals higher expression of immune suppression checkpoint proteins, CD274, PDCD1, CTLA4, and LAG3, in the low-risk group. Moreover, the low-risk group showed higher values for all four immunotherapy scores than the high-risk group. TMB is the total number of somatic mutations found in a tumor sample, a score which can influence activation of the body's antitumor response [14]. The findings of the current study indicate a negative association between TMB and OS. The correlation of TMB and model grouping revealed that the low-risk group combined with low TMB scores had the best survival whereas the high-risk group combined with high TMB scores had the worst survival. To date, few studies link TMB to predictions of the efficacy of ICI drugs in BC, although TMB is closely related to BC prognosis and can be used as a prognostic indicator. In ER+ BC, high TMB is usually associated with a poor prognosis [32]. Thus, we infer that our prediction model may reveal reliable immune biomarkers for BC treatment, in addition to providing insights into molecular mechanisms of ferroptosis-related lncRNA in BC.

Although pathological staging and the molecular classification of cancer are widely used in a clinical context for the prognosis of BC, patients at the same stage or with the same molecular classification frequently have different clinical outcomes, indicating a lack of reliability of this system [33]. Therefore, further work is necessary to identify new potential predictive and therapeutic biomarkers. The ferroptosis-related lncRNA model that we have described addresses this need and also reveals insights into molecular mechanisms of ferroptosis. Although we have verified our model using a variety of methods, we are aware that shortcomings and limitations remain. The lack of verification using external databases and the absence of stratification analysis according to the molecular type of BC may lead to bias in the results. In future studies, we plan to collect further clinical samples and integrate multicenter studies to verify the accuracy of our model while continuing to explore the role of lncRNA and its interaction with ferroptosis.

In conclusion, the specific ferroptosis-related lncRNAs identified in the current study have utility in assessing BC prognosis and may illuminate the roles of lncRNA in ferroptosis mechanisms. Our predictive model shows great promise as a sensitive predictor of BC patient response to immunotherapy.

## 4. Materials and Methods

### 4.1. Data Collection from Patients with Invasive BC

We collated RNA sequence, clinical information, and mutation incidence data from invasive BC patients (112 normal and 1,096 tumor samples) from the TCGA-BRCA project (https://cancergenome.nih.gov/). We excluded patients whose follow-up time was less than 30 days.

### 4.2. Identification of Ferroptosis-Related Genes and lncRNAs

Data analysis was performed by R software (version 4.0.3), Perl 5 (version 5.32.1), and GSEA (gene set enrichment analysis) (version 4.1.0). We used a ferroptosis-related gene list from FerDb (http://www.zhounan.org/ferrdb/) to identify ferroptosis-related genes. Using a screening based on Pearson's correlation analysis (threshold of ∣*R*^2^ | >0.4; *p* < 0.001), we were able to identify 1,636 ferroptosis-related lncRNAs.

### 4.3. Differential Expression Analysis of Ferroptosis-Related lncRNA

We used the R package, limma, to analyze the differential expression of ferroptosis-related lncRNAs. The screening threshold was set at FDR < 0.05, and ∣log2FC | ≥1.299 differentially expressed ferroptosis-related lncRNAs were identified.

### 4.4. Construction and Verification of Risk Model

We randomly divided the TCGA-BRCA dataset into two equal groups: a training set and a test set. The training set was used to construct a ferroptosis-related lncRNA risk model, and the test set was used to verify the model. Using BC patient survival information, we related the differential expression of 299 ferroptosis-related lncRNAs to prognosis (*p* < 0.05). We performed univariate, LASSO, and multifactor Cox regression to construct a risk model [34–36]. We were able to establish a risk model based on the expression of 10 ferroptosis-related lncRNAs. The risk score could be calculated according to the following formula:

(1) $$\begin{array}{c} \text{Risk score}=\sum \text{c}\text{oefficient }\left(\text{gene }i\right)*\text{expression value }\left(\text{gene }i\right). \end{array}$$

Median risk scores allowed the patients to be divided into the high- and low-risk groups [37].

### 4.5. Enrichment Analysis of Ferroptosis-Related Genes and lncRNAs

We performed Gene Ontology (GO) and Kyoto Encyclopedia of Genes and Genomes (KEGG) analysis of ferroptosis-related genes using the R package, BiocManager. The risk model was further enriched and analyzed by GSEA [38]. The analysis threshold was set according to the adjusted *p* value (*q* value) < 0.05.

### 4.6. Principal Component and Kaplan-Meier Survival Analysis

We used principal component analysis (PCA) to reduce the dimensionality, model identification, and group visualization of total genes, ferroptosis-related genes, and ferroptosis-related lncRNAs, including the ten lncRNAs identified as contributing to the risk model [39]. We used Kaplan-Meier survival analysis to assess differences in overall survival (OS) between the high- and low-risk groups. The R packages used in the above analysis were limma, scatterplot3d, survival, and survminer.

### 4.7. Correlation of the Model with Immune Function

To assess immune cell infiltration in the high- and low-risk groups, we applied software (TIMER, CIBERSORT, XCELL, QUANTISEQ, MCPcounter, EPIC, and CIBERSORT-ABS) to the TCGA-BRCA database [40]. Single-sample gene set enrichment analysis (ssGSEA) was used to assess differences in immune function between patients in the high- and low-risk groups. In addition, we analyzed relevant immune suppression checkpoints and used immunotherapy antigen to calculate the four types of immunophenoscore (IPS) for BC patients [41]. The R packages used were *scales*, *ggplot2*, *ggtext*, *pheatmap*, *GSVA*, *GSEABase*, *ggpubr*, and *reshape2*.

### 4.8. Correlation of the Model with TMB

We used the R package, *maftools*, to analyze mutation data. Patients with different TMB scores were divided into subgroups for survival analysis. TMB was also correlated with the risk model.

### 4.9. Prediction of Treatment Sensitivity of Patients with Different Risk Scores

To derive potential compounds for the clinical treatment of BC, the IC50 for compounds obtained from the Genomics of Drug Sensitivity in Cancer (GDSC) website was calculated for the TCGA-BRCA project. The R package *pRRophetic* was used to predict the IC50 of the compound obtained from the GDSC website in BC patients.

### 4.10. Independent Verification of the Ferroptosis-Related lncRNA Model

Our risk model was analyzed using single- and multiple-factor Cox regression analyses to evaluate its suitability as an independent prognostic factor by comparison with classic clinicopathological characteristics of BC patients.

### 4.11. Construction and Verification of Nomogram

A nomogram integrating the prognostic signature was constructed to predict the 1-, 3-, and 5-year OS of BC patients. In addition, predictions were calibrated using the Hosmer-Lemeshow test to evaluate accuracy. The R packages used were *regplot*, *survival*, and *rms*.

### 4.12. Cell Lines

Breast cancer cell lines (MCF7, SKBR3, and MDA-MB-231) and a normal human breast epithelial cell line (MCF10A) were bought from the Type Culture Collection of the Chinese Academy of Sciences (Beijing, China). These cell lines were cultured with DMEM (Gibco) supplemented with 10% certified fetal bovine serum (VivaCell, Shanghai, China).

### 4.13. RNA Isolation and Quantitative Real-Time PCR (qRT-PCR)

Total RNA was isolated using the TRIzol reagent following the manufacturer's protocol (Takara, Japan) and reversely transcribed into cDNA using PrimeScript RT Master Mix (Takara), after which expression of the target gene was evaluated by qRT-PCR using SYBR Green Mix (Takara) according to the manufacturer's instructions. The primers used were listed as follows: CYTOR: 5′-ACAGACACCGAAAATCACGACT-3′ (forward), 5′-CAGGCAGACCACCCGCAAA-3′ (reverse); LMNTD2-AS1: 5′-CAGCGCACGTTAACCTCGAA-3′ (forward), 5′-TGCCTGTAGTTTCAGCAAGTCA-3′ (reverse); LYPLAL1-AS1: 5′-AGAGTCCCCACCAGCAAGAAG-3′ (forward), 5′-CTCCACACAATCTGTCCCGAA-3′ (reverse); USP30-AS1: 5′-TTTTCAGATTTGCTTAGGCTCCA-3′ (forward), 5′-CTCCCTTCCCCACATCGAA-3′ (reverse); RHPN1-AS1: 5′-CTCGCCTCAGCTCAGAACACA-3′ (forward), 5′-ACAGGCACCAGAATGATCCCA-3′ (reverse); HSD11B1-AS1: 5′-CCATACCAAATCCAACGCCTA-3′ (forward), 5′-ACACTTCAGCTCTTTGCACT-3′ (reverse); LINC01655: 5′-TCCTTGTTCTGTCACCAAGCCTT-3′ (forward), 5′-GTGCAGATCCTGACCCCTT-3′ (reverse); AP005131.2: 5′-GTCAGATTGCCAATGGTTCCT-3′ (forward), 5′-AGATGATATGCCACAACACGAA-3′ (reverse); AC004988.1: 5′-ATGTCTCCCTTTAGTGCCAGT-3′ (forward), 5′-GCACATTCAGAAATGACCTCGAA-3′ (reverse); and AC079298.3: 5′-TGAGAGGACCATTTTCTGACTGT-3′ (forward), 5′-TCCTTTCATCCAGGGTGGTGTT-3′ (reverse).

Results were normalized using GAPDH, and the 2−*ΔΔ*CT method was applied to analyze the expression of target genes.

## Data Availability

The data used to support the findings of this study are available from the corresponding author upon request.

## Supplementary Materials

Supplementary 1 Figure S1: flow chart of this study.

Supplementary 2 Figure S2: principal component analysis between the high- and low-risk groups based on (A) total gene expression profile, (B) ferroptosis-related genes, (C) ferroptosis-related lncRNAs, and (D) risk model based on the representation profiles of the 10 ferroptosis-related lncRNAs in the TCGA-BRCA entire set.

Supplementary 3 Figure S3: gene enrichment analysis for the ferroptosis-related lncRNAs of the low-risk group based on the TCGA-BRCA entire set.

Supplementary 4 Figure S4: identification of novel candidate compounds targeting ferroptosis-related lncRNA models.

Supplementary 5 Figure S5: the relative expression of CYTOR, LMNTD2-AS1, LYPLAL1-AS1, USP30-AS1, RHPN1, LINC01655, AP005131.2, AC004988.1, HSD11B1-AS1, and AC079289.3 was confirmed by qPCR (*n* = 10).

Supplementary 6 Table S1: ferroptosis-related genes were identified from FerDb (http://www.zhounan.org/ferrdb/).

Supplementary 7 Table S2: ferroptosis-related lncRNAs were differentially expressed.

Supplementary 8 Table S3: ten ferroptosis-related lncRNA risk model.

Supplementary 9 Table S4: gene enrichment analysis for ferroptosis-related lncRNAs based on the TCGA-BRCA entire set.
