# Supplementary figures and images for: A Novel Prognostic Ferroptosis-Related lncRNA Signature Associated with Immune Landscape in Invasive Breast Cancer
Source: Dis Markers. 2022 Mar 20;2022:9168556. doi: 10.1155/2022/9168556 (PMC8961446; doi:10.1155/2022/9168556)

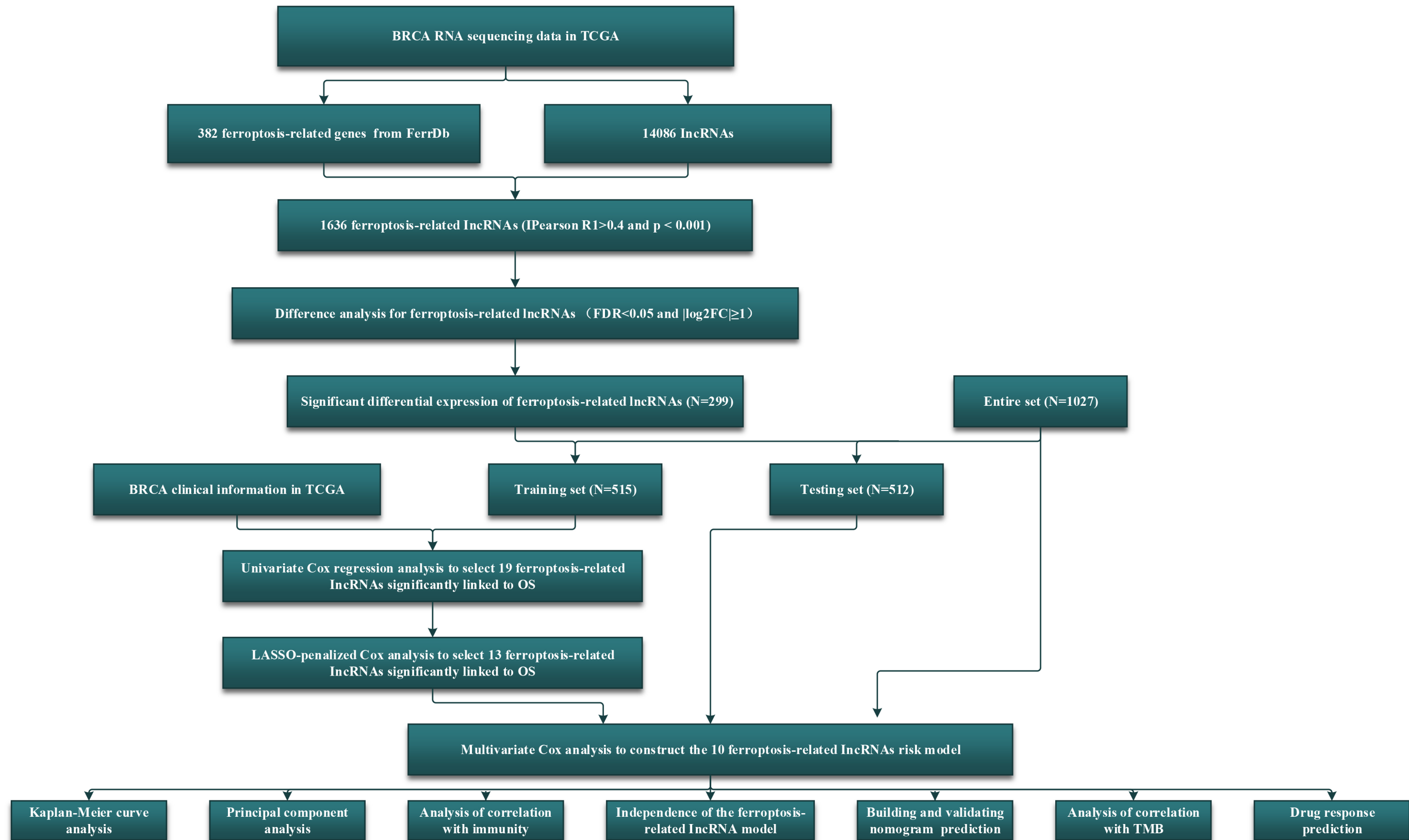

Supplement: Supplementary 1 — Figure S1: flow chart of this study. [file 9168556.f1.pdf]

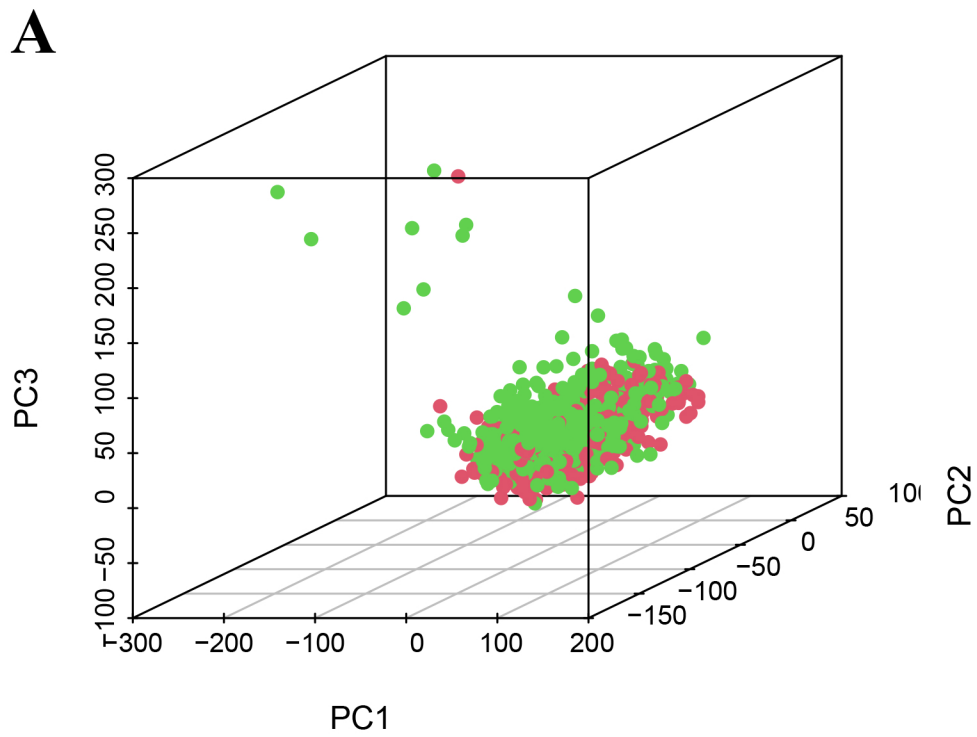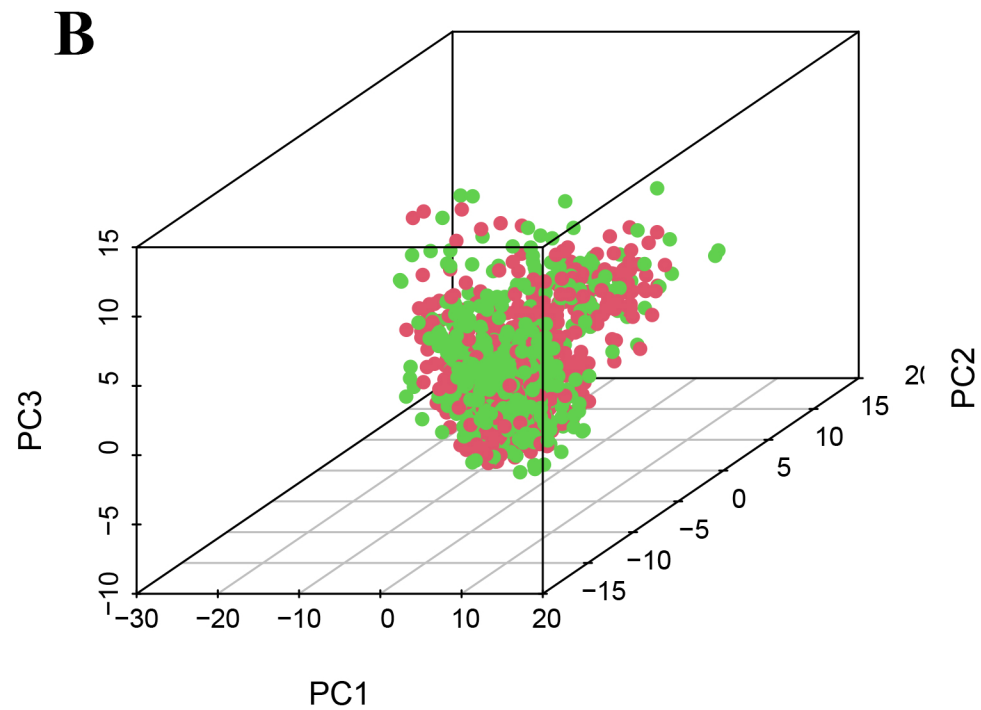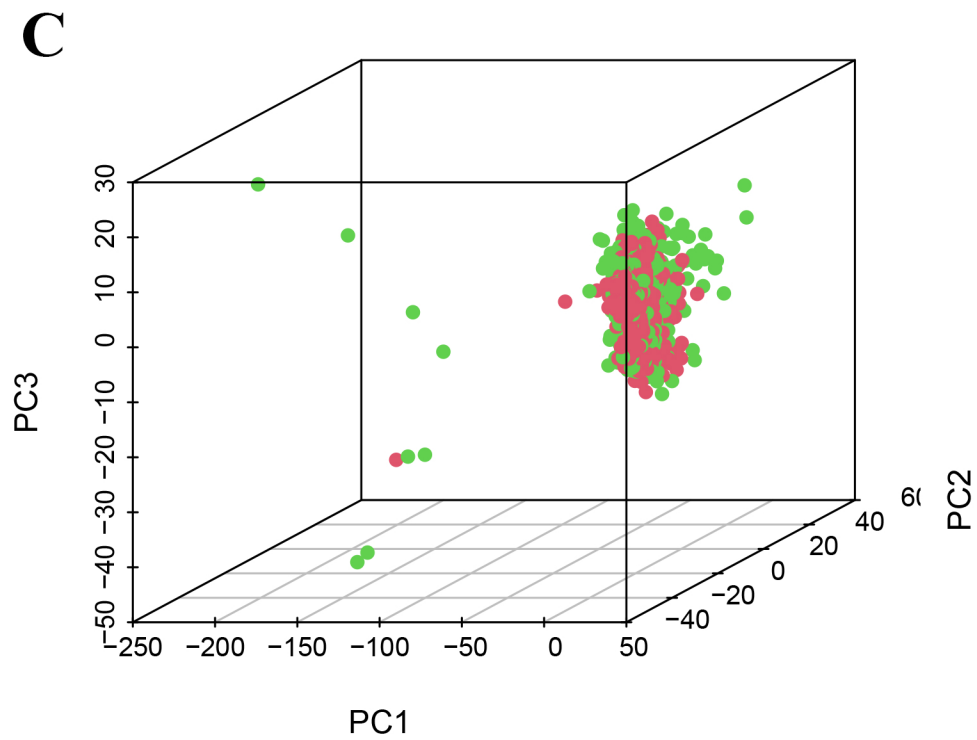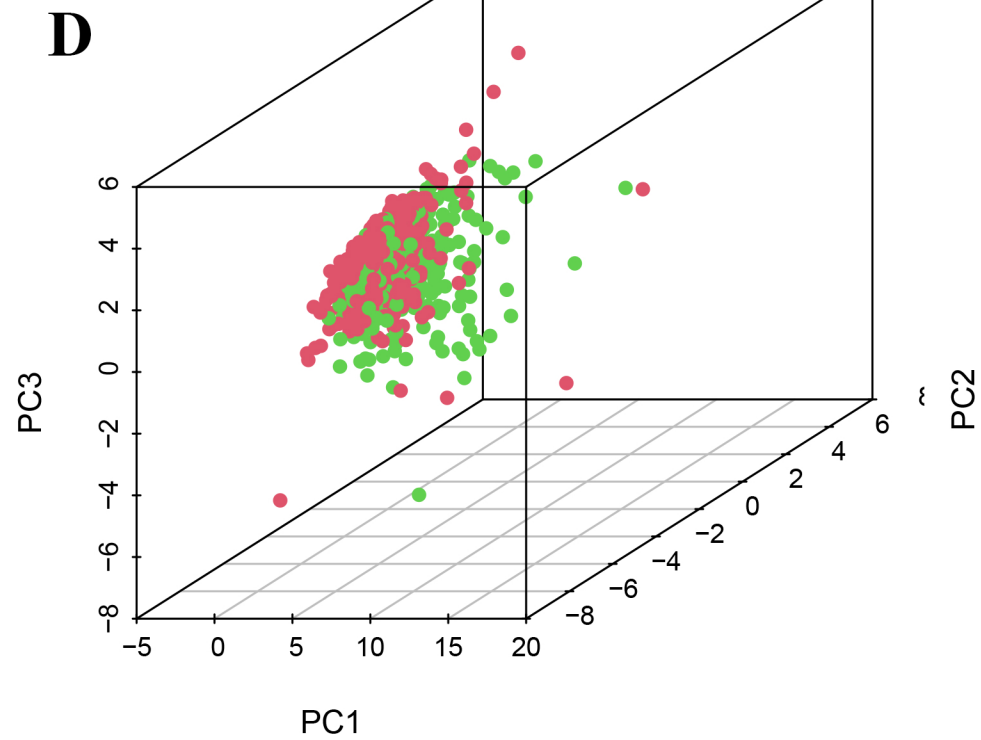

Supplement: Supplementary 2 — Figure S2: principal component analysis between the high- and low-risk groups based on (A) total gene expression profile, (B) ferroptosis-related genes, (C) ferroptosis-related lncRNAs, and (D) risk model based on the representation profiles of the 10 ferroptosis-related lncRNAs in the TCGA-BRCA entire set. [file 9168556.f2.pdf]

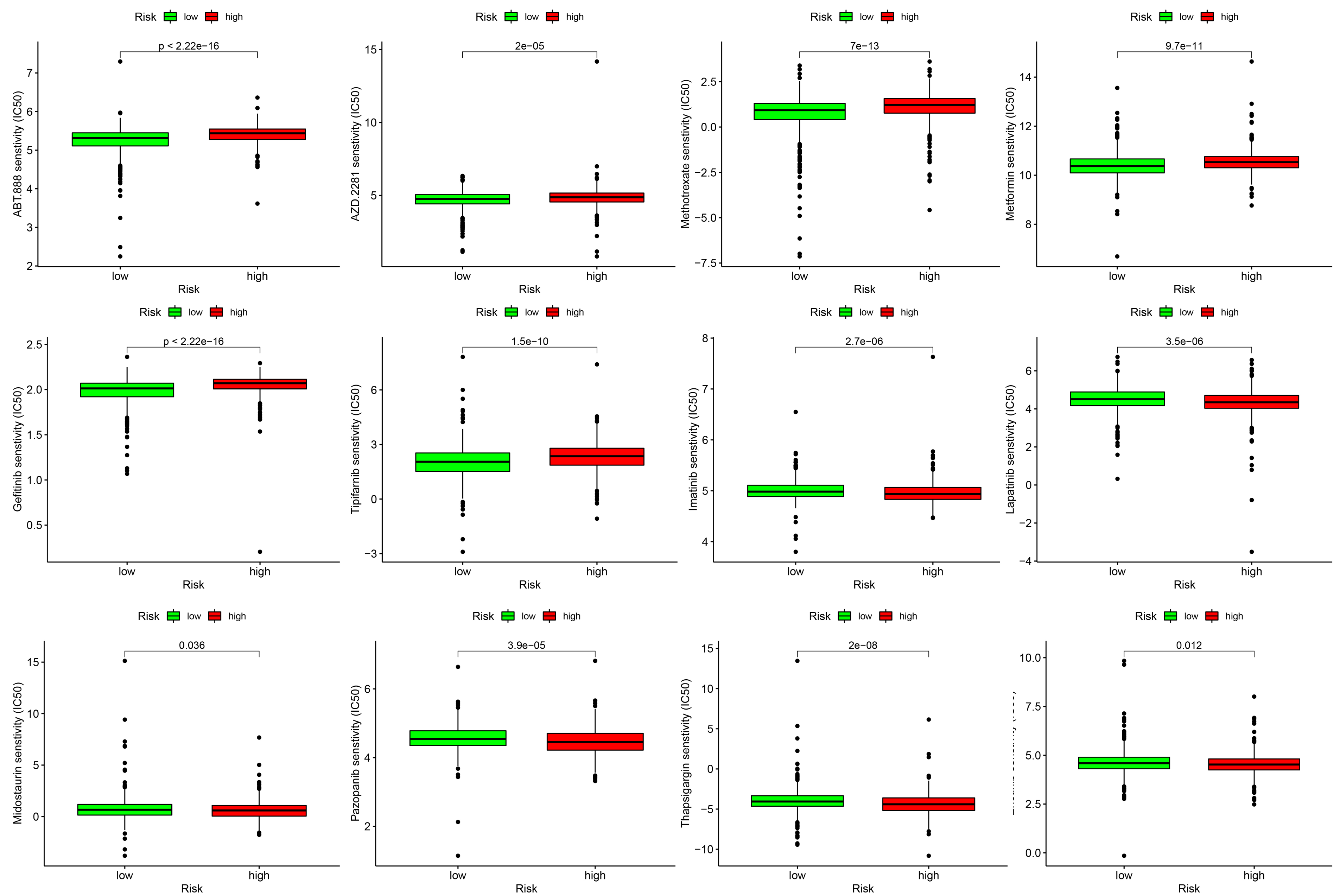

Supplement: Supplementary 4 — Figure S4: identification of novel candidate compounds targeting ferroptosis-related lncRNA models. [file 9168556.f4.pdf]

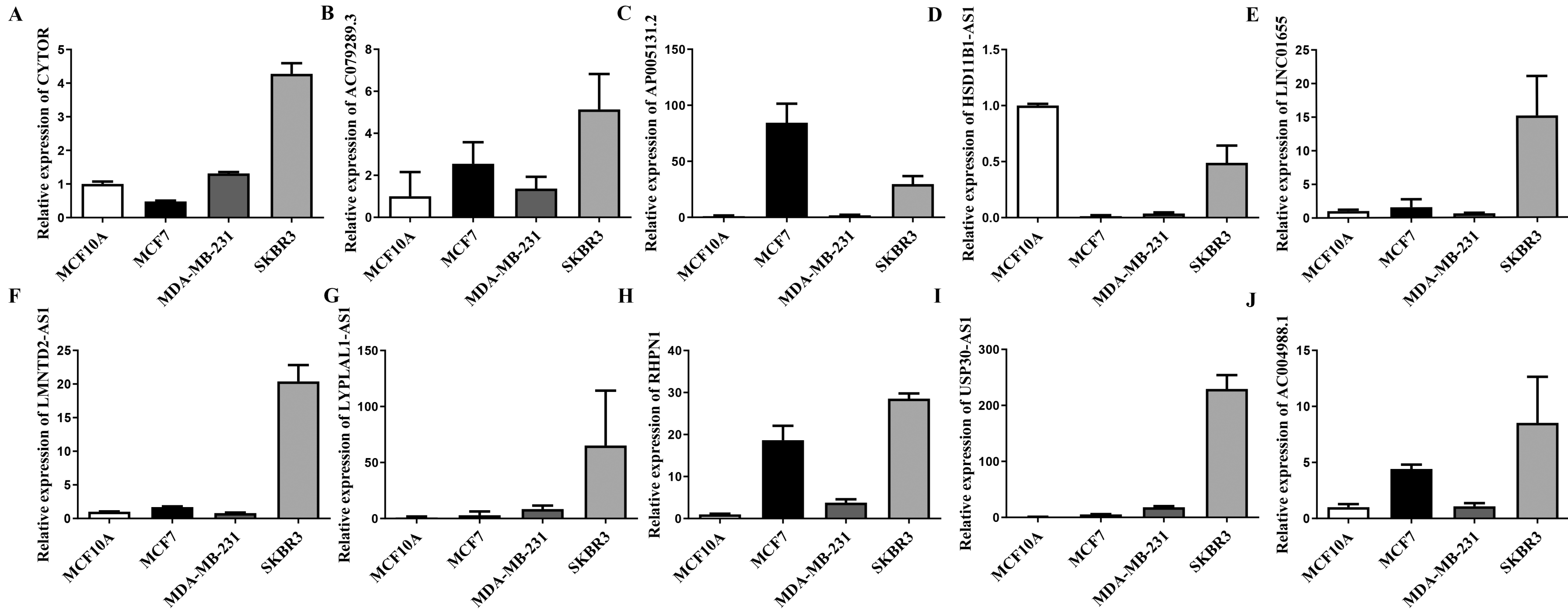

Supplement: Supplementary 5 — Figure S5: the relative expression of CYTOR, LMNTD2-AS1, LYPLAL1-AS1, USP30-AS1, RHPN1, LINC01655, AP005131.2, AC004988.1, HSD11B1-AS1, and AC079289.3 was confirmed by qPCR (n = 10). [file 9168556.f5.pdf]
