# Supplementary material for: A Novel Prognostic Ferroptosis-Related lncRNA Signature Associated with Immune Landscape in Invasive Breast Cancer
Source: Dis Markers. 2022 Mar 20;2022:9168556. doi: 10.1155/2022/9168556 (PMC8961446; doi:10.1155/2022/9168556)
Supplement: Supplementary 6 — Table S1: ferroptosis-related genes were identified from FerDb (http://www.zhounan.org/ferrdb/). [file 9168556.f6.pdf]

Table S1:Ferroptosis-related genes were identified from FerDb(http://www.zhounan.

Symbol	Name	HGNC_ID	Evidence	Test meth	Test in	Test settin	Pathway	Confidenc
RPL8	Ribosomal	HGNC:103	Required i	shRNA scr	Human	NRAS mut	RPL8 :+: F	Validated
IREB2	Iron respo	HGNC:611	Required i	shRNA scr	Human	NRAS mut	IREB2 :+: F	Validated
ATP5MC3	ATP synth	HGNC:843	Required i	shRNA scr	Human	NRAS mut	ATP5MC3	Validated
CS	Citrate syr	HGNC:242	Required i	shRNA scr	Human	NRAS mut	CS :+: Ferr	Validated
EMC2	ER membr	HGNC:289	Required i	shRNA scr	Human	NRAS mut	EMC2 :+: F	Validated
ACSF2	Acyl-CoA	HGNC:261	Required i	shRNA scr	Human	NRAS mut	ACSF2 :+:	Validated
NOX1	Nicotinam	HGNC:788	Suppresse	Inhibition	Human	KRAS mut	NOX1 :+: I	Deduced
CYBB	Cytochron	HGNC:257	Suppresse	Inhibition	Human	KRAS mut	CYBB :+: F	Deduced
NOX3	Nicotinam	HGNC:789	Suppresse	Inhibition	Human	KRAS mut	NOX3 :+: I	Deduced
NOX4	Nicotinam	HGNC:789	Suppresse	Inhibition	Human	KRAS mut	NOX4 :+: I	Deduced
NOX5	Nicotinam	HGNC:148	Suppresse	Inhibition	Human	KRAS mut	NOX5 :+: I	Deduced
DUOX1	Dual oxida	HGNC:306	Suppresse	Inhibition	Human	KRAS mut	DUOX1 :+	Deduced
DUOX2	Dual oxida	HGNC:132	Suppresse	Inhibition	Human	KRAS mut	DUOX2 :+	Deduced
G6PD	Glucose-6	HGNC:405	Required i	shRNA sile	Human	KRAS mut	G6PD :+: F	Validated
PGD	Phosphog	HGNC:889	Required i	shRNA sile	Human	KRAS mut	PGD :+: Fe	Validated
VDAC2	Valtage-d	HGNC:126	Required i	shRNA sile	Human	KRAS mut	VDAC2 :+:	Validated
PIK3CA	Phosphati	HGNC:897	Inhibitor b	Cell surviv	Mice	HT22 cells	PIK3CA :-:	Validated
FLT3	Fms relate	HGNC:376	Inhibitor b	Cell surviv	Mice	HT22 cells	FLT3 :+: Li	Validated
SCP2	Sterol carr	HGNC:106	SCP-2 inh	Cell viabili	Mice	PZL cells	SCP2 :+: F	Validated
TP53	Tumor pro	HGNC:119	Inhibits cy	Cell death	Human, m	H1299, U2	TP53 :-: Sl	Validated
ACSL4	Acyl-CoA	HGNC:357	Deletion o	Retroviral-	Human	Chronic m	ACSL4 :+:	Predicted
LPCAT3	Lysophosp	HGNC:302	Deletion o	Retroviral-	Human	Chronic m	LPCAT3 :+	Predicted
NRAS	NRAS prot	HGNC:798	NRAS12V	Cell viabili	Human	RMS13 cel	NRAS :+: F	Deduced
KRAS	KRAS prot	HGNC:640	KRAS12V	Cell viabili	Human	RMS13 cel	KRAS :+: F	Deduced
HRAS	HRas prot	HGNC:517	HRAS12V	Cell viabili	Human	RMS13 cel	HRAS :+: F	Deduced
TF	Transferrir	HGNC:117	Essential f	Killing acti	Mice	Mouse em	TF :+: Ferr	Validated
TFRC	Transferrir	HGNC:117	RNAi of tr	RNAi	Mice	Mouse em	TFRC :+: F	Validated
TFR2	Transferrir	HGNC:117	RNAi of tr	RNAi	Mice	Mouse em	TFR2 :+: F	Validated
SLC38A1	Solute carr	HGNC:134	RNAi knoc	RNAi knoc	Mice	Mouse em	SLC38A1 :	Validated
SLC1A5	Solute carr	HGNC:109	Pharmaco	RNAi knoc	Mice	Mouse em	SLC1A5 :+	Validated
GLS2	Glutamina	HGNC:295	Both inhib	shRNA, we	Mice	Mouse em	GSL2 :+: G	Validated
GOT1	Glutamic-	HGNC:443	RNAi redu	shRNA, qP	Mice	Mouse em	GOT1 :+: a	Validated
CARS1	Cysteinyl-	HGNC:149	Required f	siRNA scre	Human, ra	HT-1080 f	CARS1 :-:	Validated
TP53	Tumor pro	HGNC:119	Wild type	Cell death	Human	H1299 cel	TP53 :-: Sl	Validated
ALOX5	Arachidon	HGNC:435	The 5-Lip	Cell Viabili	Mice	HT22 mou	ALOX5 :+:	Validated
KEAP1	Kelch like	HGNC:231	Knockdow	Western b	Human, m	HepG2, He	KEAP1 :-: I	Validated
HMOX1	Heme oxy	HGNC:501	Zinc proto	Cell viabili	Human, m	HT-1080 f	HMOX1 :+	Validated
TP53	Tumor pro	HGNC:119	p53^3KR/	Western b	Mice	p53^3KR/	TP53 :-: Sl	Validated
TP53	Tumor pro	HGNC:119	Incubation	Cell viabili	Human, m	Mouse em	TP53 :-: Sl	Validated
GLS2	Glutamina	HGNC:295	Upregulat	Cell viabili	Mice	Mouse em	GLS2 :+: F	Validated
ATG5	Autophag	HGNC:589	Knockout	shRNA	Human, m	Mouse em	ATG5 :+: F	Validated

ATG7	Autophag	HGNC:169	Knockout	shRNA	Human, m	Mouse em	ATG7 :+: F	Validated
NCOA4	Nuclear re	HGNC:767	Inhibition	shRNA, ge	Human	PANC1 or	NCOA4 :-:	Validated
TF	Transferrir	HGNC:117	Involved ir	Western b	Human	MDA MB 2	TF :+: Lipi	Validated
ALOX5	Arachidon	HGNC:435	Silencing A	qPCR	Human	G-401 cell	ALOX5 :+:	Validated
ALOX12	Arachidon	HGNC:429	Silencing A	qPCR	Human	G-401 cell	ALOX12 :+	Validated
ALOX12B	Arachidon	HGNC:430	Silencing A	qPCR	Human	G-401 cell	ALOX12B	Validated
ALOX15	Arachidon	HGNC:433	Silencing A	qPCR	Human	G-401 cell	ALOX15 :+	Validated
ALOX15B	Arachidon	HGNC:434	Silencing A	qPCR, siRN	Human	G-401, BJe	ALOX15B	Validated
ALOXE3	Arachidon	HGNC:137	Silencing A	qPCR, siRN	Human	G-401, BJe	ALOXE3 :+	Validated
PHKG2	Phosphory	HGNC:893	U-2-OS ce	shRNA sup	Human	U-2-OS, H	PHKG2 :+:	Validated
TFRC	Transferrir	HGNC:117	The gene t	RNAi scree	Mice	Mouse em	TFRC :+: L	Screened
ACO1	Aconitase	HGNC:117	The gene t	RNAi scree	Mice	Mouse em	ACO1 :+: I	Screened
IREB2	iron respo	HGNC:611	The gene t	RNAi scree	Mice	Mouse em	IREB2 :+: L	Screened
SLC38A1	Solute carr	HGNC:134	The gene t	RNAi scree	Mice	Mouse em	SLC38A1 :	Screened
GLS2	Glutamina	HGNC:295	The gene t	RNAi scree	Mice	Mouse em	GLS2 :+: Li	Screened
G6PDX	_NA_	_NA_	The gene t	RNAi scree	Mice	Mouse em	G6PDX :+:	Screened
ULK1	Unc-51 lik	HGNC:125	Potential p	RNAi scree	Mice	Mouse em	ULK1 :+: L	Validated
ATG3	Autophag	HGNC:209	Potential p	RNAi scree	Mice	Mouse em	ATG3 :+: L	Validated
ATG4D	Autophag	HGNC:207	Potential p	RNAi scree	Mice	Mouse em	ATG4D :+:	Screened
ATG5	Autophag	HGNC:589	Potential p	RNAi scree	Mice	Mouse em	ATG5 :+: L	Validated
BECN1	Beclin 1	HGNC:103	Potential p	RNAi scree	Mice	Mouse em	BECN1 :+:	Screened
MAP1LC3A	Microtubu	HGNC:683	Potential p	RNAi scree	Mice	Mouse em	MAP1LC3A	Screened
GABARAP	GABA type	HGNC:132	Potential p	RNAi scree	Mice	Mouse em	GABARAP	Screened
GABARAP	GABA type	HGNC:406	Potential p	RNAi scree	Mice	Mouse em	GABARAP	Screened
ATG16L1	Autophag	HGNC:214	Potential p	RNAi scree	Mice	Mouse em	ATG16L1 :	Screened
WIPI1	WD repea	HGNC:254	Potential p	RNAi scree	Mice	Mouse em	WIPI1 :+: L	Screened
WIPI2	WD repea	HGNC:322	Potential p	RNAi scree	Mice	Mouse em	WIPI2 :+: L	Screened
SNX4	Sorting ne	HGNC:111	Potential p	RNAi scree	Mice	Mouse em	SNX4 :+: L	Screened
ATG13	Autophag	HGNC:290	Knockout	Gene knoc	Mice	Mouse em	ATG13 :+:	Validated
ULK2	Unc-51 lik	HGNC:134	Knockout	Gene knoc	Mice	Mouse em	ULK2 :+: L	Validated
NCOA4	Nuclear re	HGNC:767	Eliminatior	RNAi knoc	Human, m	Mouse em	NCOA4 :+	Validated
ACSL4	Acyl-CoA	HGNC:357	Knockdow	shRNA, ge	Human	LNCaP (hu	ACSL4 :+:	Validated
TP53	Tumor pro	HGNC:119	p53 acetyl	Mass spec	Human, m	H1299 cell	TP53 :-: SL	Validated
SAT1	Spermidin	HGNC:105	p53-media	qRT-PCR,	Human, m	H1299, U2	SAT1 :+: A	Validated
ALOX15	Arachidon	HGNC:433	SAT1- anc	Cell death	Human	H1299, U2	ALOX15 :+	Validated
ACSL4	Acyl-CoA	HGNC:357	Inhibition	Cell death	Mice	Mouse em	ACSL4 :+:	Validated
LPCAT3	Lysophosp	HGNC:302	Knockdow	shRNA	Mice	Mouse lur	LPCAT3 :+	Validated
ALOX15	Arachidon	HGNC:433	Liproxstati	Identificati	Mice	Kidney of	ALOX15 :+	Validated
ACSL4	Acyl-CoA	HGNC:357	An essenti	Cell viabili	Human, m	Mouse em	ACSL4 :+:	Validated
KEAP1	Kelch like	HGNC:231	Keap 1 sile	Cell viabili	Mice	Head and	KEAP1 :-:	Validated
EGFR	Epidermal	HGNC:323	Cell death	Fluorescer	Human	Human ma	EGFR :+: M	Validated
NOX4	NADPH ox	HGNC:789	Inhibition	Fluorescer	Human	Human ma	NOX4 :+: I	Validated
MAPK3	Mitogen-a	HGNC:687	Inhibiting	Immunobl	Human	Human ma	MAPK :-: (	Validated

MAPK1	Mitogen-a	HGNC:687	Inhibiting	Immunobl	Human	Human ma	MAPK :-: (	Validated
BID	BH3 intera	HGNC:105	BID deletic	Cell viabili	Mice	HT-22 cell	BID :+: Fer	Validated
ACSL4	Acyl-CoA	HGNC:357	Knockout	CRISPR/Ca	Human, m	NIH3T3, H	ACSL4 :+:	Validated
ZEB1	Zinc finger	HGNC:116	Knockout	sgRNA, ce	Human	KP4 pancr	ZEB1 :+: Li	Validated
KEAP1	Kelch like	HGNC:231	Keap1 inhi	Cell viabili	Human, ra	F98, U87 g	KEAP1 :+:	Validated
DPP4	Dipeptidyl	HGNC:300	Required f	Cell death	Human, m	TP53-/- H	DPP4 :+: L	Validated
ALOX15	Arachidon	HGNC:433	Suppressic	siRNA, trai	Human	HT1080, P	ALOX15 :+	Validated
ALOX12	Arachidon	HGNC:429	12-LOX in	siRNA, trai	Human	HT1080, P	ALOX12 :+	Validated
CDKN2A	Cyclin dep	HGNC:178	Combinati	RNAi, imm	Human, m	H1299, Sa	CDKN2A :	Validated
PEBP1	Phosphati	HGNC:863	Elevated le	Western b	Human, m	HK2, HAE(	(PEBP1/15	Validated
SOCS1	Suppresso	HGNC:193	Expressior	Cell death	Human	U2OS or II	SOCS1 :+:	Validated
CDO1	Cysteine d	HGNC:179	CDO1 sup	siRNA, we	Human	Gastric car	CDO1 :-: (	Validated
MYB	MYB protc	HGNC:754	Erastin-inc	Western b	Human	Gastric car	MYB :+: Cl	Validated
HMOX1	Heme oxy	HGNC:501	Inhibiting	Cell viabili	Human	MDA-MB-	HMOX1 :+	Validated
MAPK8	Mitogen-a	HGNC:688	JNK1/2 inh	MitoSOX p	Rat	PC12 cells	MAPK8 :+	Validated
MAPK9	Mitogen-a	HGNC:688	JNK1/2 inh	MitoSOX p	Rat	PC12 cells	MAPK9 :+	Validated
MAPK1	Mitogen-a	HGNC:687	ERK1/2 inh	MitoSOX p	Rat	PC12 cells	MAPK1 :+	Validated
MAPK3	Mitogen-a	HGNC:687	ERK1/2 inh	MitoSOX p	Rat	PC12 cells	MAPK3 :+	Validated
SLC1A5	Solute carr	HGNC:109	Overexpre	Cell viabili	Human	Melanoma	SLC1A5 :+	Validated
CHAC1	ChaC gluta	HGNC:286	CHAC1 de	Western b	Human	MDA-MB-	CHAC1 :-:	Validated
MAPK14	Mitogen-a	HGNC:687	Ferroptosi	Cell viabili	Mice	TM4 Serto	MAPK14 :-	Validated
LINC0047	Long inter	HGNC:213	Increases	Bisulfite se	Human	Lung canc	LINC0047	Validated
NOX4	NADPH ox	HGNC:789	Activated	Western b	Human, ra	Rat C6, hu	NOX4 :+: I	Validated
GOT1	Glutamic-	HGNC:443	Overexpre	Immunobl	Human	A375 and	GOT1 :+: a	Validated
BECN1	Beclin 1	HGNC:103	Knockdow	shRNA, we	Human, m	HCT116, C	BECN1 :-:	Validated
PRKAA2	Protein kir	HGNC:937	Inhibition	siRNA, wes	Human	HCT116 ar	PRKAA2 :+	Validated
PRKAA1	Protein kir	HGNC:937	Inhibition	siRNA, wes	Human	HCT116 ar	PRKAA1 :+	Validated
ELAVL1	ELAV like	HGNC:331	ELAVL1 siR	Immunohi	Human, m	Human liv	ELAVL1 :+	Validated
BAP1	BRCA1 ass	HGNC:950	Suppresse	Flow cytor	Human	HEK-293T	BAP1 :-: S	Validated
TP53	Tumor prc	HGNC:119	Facilitates	Western b	Rat	Hepatic st	TP53 :+: F	Validated
ABCC1	ATP bindir	HGNC:51	Accelerate	Western b	Human	HAP1, H12	ABCC1 :-:	Validated
ACSL4	Acyl-CoA	HGNC:357	Inhibition	siRNA, wes	Human, m	Male C57E	ACSL4 :+:	Validated
MIR6852	microRNA	HGNC:499	Promotes	qRT-PCR,	Human	A549 and	MIR6852 :	Validated
ACVR1B	Activin A r	HGNC:172	Inhibition	Western b	Human	HK-2 cells	ACVR1B :-	Validated
TGFBR1	Transform	HGNC:117	Inhibition	Western b	Human	HK-2 cells	TGFBR1 :-	Validated
BAP1	BRCA1 ass	HGNC:950	Promotes	Immunopr	Human	HEK293T c	BAP1 :-: S	Validated
EPAS1	Endothelia	HGNC:337	A driver of	CRISPR, sg	Mice	786-O cel	EPAS1 :+:	Validated
HILPDA	Hypoxia in	HGNC:288	Promotes	RNA-Seq,	Mice	786-O cel	HILPDA :+	Validated
HIF1A	Hypoxia in	HGNC:491	Re-sensitiz	cDNA scre	Mice	786-O cel	HIF1A :+: I	Validated
ALOX12	Arachidon	HGNC:429	An essenti	RNAi, qRT	Human, m	H1299 cell	ALOX12 :+	Validated
ACSL4	Acyl-CoA	HGNC:357	Required f	Western b	Human	U2OS	ACSL4 :+:	Validated
HMOX1	Heme oxy	HGNC:501	Enhances	Gene trans	Human	H1299 cell	HMOX1 :+	Validated
IFNG	Interferon	HGNC:543	Interferon	BODIPY-C	Human, m	HT-1080,	IFNG :-: Sl	Validated

ANO6	Anoctamir	HGNC:252	Essential fc	Immunocy	Human, m	A549, Cal2	ANO6 :+:	Validated
LPIN1	Lipin 1	HGNC:133	Overexpre	Enzyme-li	Mice	C57BL/6 n	LPIN1 :+: I	Validated
HMGB1	High mobi	HGNC:498	Required f	Gene trans	Human, m	HL-60 cell	HMGB1 :+	Validated
TNFAIP3	TNF alpha	HGNC:118	Overexpre	Lentivirus	Human	HUVEC ce	TNFAIP3 :-	Validated
TLR4	Toll like re	HGNC:118	Knockdow	Immunohi	Rat	Sprague D	TLR4 :+: F	Validated
NOX4	NADPH ox	HGNC:789	Knockdow	Immunohi	Rat	Sprague D	NOX4 :+: I	Validated
ATF3	Activating	HGNC:785	Promotes	Cell viabili	Human	HT1080 ce	ATF3 :-: SI	Validated
ATM	ATM serin	HGNC:795	Essential fc	siRNA, we:	Human	MDA-MB-	ATM :-: M	Validated
YY1AP1	YY1 associ	HGNC:309	Makes cell	Cell death	Human, m	Mouse em	YAP :+: AC	Validated
EGLN2	Egl-9 fami	HGNC:146	Inhibiting	Cytotoxicit	Human, m	Calu-1, TH	EGLN2 :-:	Validated
MIOX	Myo-inosi	HGNC:145	Overexpre	MTT assay	Human, m	HK-2 cells	MIOX :-: C	Validated
TAZ	Tafazzin	HGNC:115	TAZ remo	Cell viabili	Human, m	RCC4, 78	TAZ :+: EN	Validated
MTDH	Metadheri	HGNC:296	Can enhar	Cell viabili	Human, m	Cell lines /	MTDH :-:	Validated
IDH1	Isocitrate	HGNC:538	Deletion o	Cell transf	Human	HEK293T,	IDH1 :-: G	Validated
SIRT1	Sirtuin 1	HGNC:149	Knockout	Lipid pero:	Mice	Mice in C5	SIRT1 :+: F	Predicted
TAZ	Tafazzin	HGNC:115	TAZ remo	siRNA, qR	Human	TOV-21G,	TAZ :+: AN	Validated
BECN1	Beclin 1	HGNC:103	Overexpre	Cell viabili	Human	SH-SY5Y c	(Beclin1/SI	Validated
FBXW7	F-box and	HGNC:167	FBXW7 pla	Cell viabili	Human, m	Hepatic st	FBXW7 :+:	Deduced
PANX1	Pannexin 1	HGNC:859	Deletion p	Cell viabili	Human, m	HK-2 cells	PANX1 :-:	Validated
DNAJB6	DnaJ heat	HGNC:148	Promotes	Western b	Human, m	Cell lines T	DNAJB6 :-	Predicted
BACH1	BTB doma	HGNC:935	Promotes	Western b	Mice	C57BL/6J	BACH1 :+:	Validated
ACSL4	Acyl-CoA	HGNC:357	Overexpre	Western b	Human	HEB, T98G	ACSL4 :-:	Validated
LONP1	Lon peptic	HGNC:947	Inhibition	Cell viabili	Human	PANC1, Bx	LONP1 :-:	Validated
PTGS2	Prostaglar	HGNC:960	Simply a d	RT-qPCR,	Human	BJeLR cells	Validated	_NA_
DUSP1	Dual speci	HGNC:306	Expressior	RT-qPCR,	Human	BJeLR cells	Deduced	Inferred as
NOS2	Nitric oxid	HGNC:787	Expressior	RT-qPCR,	Human	BJeLR cells	Deduced	Inferred as
NCF2	Neutrophi	HGNC:766	Expressior	RT-qPCR,	Human	BJeLR cells	Deduced	Inferred as
MT3	Metallothi	HGNC:740	Expressior	RT-qPCR,	Human	BJeLR cells	Deduced	Inferred as
UBC	Ubiquitin (	HGNC:124	Expressior	RT-qPCR,	Human	BJeLR cells	Deduced	Inferred as
ALB	Albumin	HGNC:399	Expressior	RT-qPCR,	Human	BJeLR cells	Deduced	Inferred as
TXNRD1	Thioredox	HGNC:124	Expressior	RT-qPCR,	Human	BJeLR cells	Deduced	Inferred as
SRXN1	Sulfiredoxi	HGNC:161	Expressior	RT-qPCR,	Human	BJeLR cells	Deduced	Inferred as
GPX2	Glutathion	HGNC:455	Expressior	RT-qPCR,	Human	BJeLR cells	Deduced	Inferred as
BNIP3	BCL2 inter	HGNC:108	Expressior	RT-qPCR,	Human	BJeLR cells	Deduced	Inferred as
OXSR1	Oxidative	HGNC:850	Expressior	RT-qPCR,	Human	BJeLR cells	Deduced	Inferred as
SELENOS	Selenopro	HGNC:303	Expressior	RT-qPCR,	Human	BJeLR cells	Deduced	Inferred as
ANGPTL7	Angiopoie	HGNC:240	Expressior	RT-qPCR,	Human	BJeLR cells	Deduced	Inferred as
CHAC1	ChaC glut	HGNC:286	Up-regula	RNA-seq,	Human	HT-1080 a	Validated	Further stu
SLC7A11	Solute carr	HGNC:110	Similar to	RNA silenc	Human	HT-1080 c	Deduced	Further stu
DDIT4	DNA dama	HGNC:249	Up-regula	RNA-seq	Human	HT-1080 c	Screened	Further stu
LOC28456	_NA_	_NA_	Up-regula	RNA-seq	Human	HT-1080 c	Screened	Further stu
ASNS	Asparagin	HGNC:753	Up-regula	RNA-seq	Human	HT-1080 c	Screened	Further stu
TSC22D3	TSC22 dor	HGNC:305	Up-regula	RNA-seq	Human	HT-1080 c	Screened	Further stu

DDIT3	DNA dama	HGNC:272	Up-regula	RNA-seq	Human	HT-1080 c	Screened	Further stu
JDP2	Jun dimeri	HGNC:175	Up-regula	RNA-seq	Human	HT-1080 c	Screened	Further stu
SESN2	Sestrin 2	HGNC:207	Up-regula	RNA-seq	Human	HT-1080 c	Screened	Further stu
SLC1A4	Solute carı	HGNC:109	Up-regula	RNA-seq	Human	HT-1080 c	Screened	Further stu
PCK2	Phosphoe	HGNC:872	Up-regula	RNA-seq	Human	HT-1080 c	Screened	Further stu
TXNIP	Thioredox	HGNC:169	Up-regula	RNA-seq	Human	HT-1080 c	Screened	Further stu
VLDLR	Very low c	HGNC:126	Up-regula	RNA-seq	Human	HT-1080 c	Screened	Further stu
GPT2	Glutamic-	HGNC:180	Up-regula	RNA-seq	Human	HT-1080 c	Screened	Further stu
PSAT1	Phosphos	HGNC:191	Up-regula	RNA-seq	Human	HT-1080 c	Screened	Further stu
LURAP1L	Leucine ric	HGNC:314	Up-regula	RNA-seq	Human	HT-1080 c	Screened	Further stu
SLC7A5	Solute carı	HGNC:110	Up-regula	RNA-seq	Human	HT-1080 c	Screened	Further stu
HERPUD1	Homocyst	HGNC:137	Up-regula	RNA-seq	Human	HT-1080 c	Screened	Further stu
XBP1	X-box bin	HGNC:128	Up-regula	RNA-seq	Human	HT-1080 c	Screened	Further stu
ATF3	Activating	HGNC:785	Up-regula	RNA-seq	Human	HT-1080 c	Screened	Further stu
SLC3A2	Solute carı	HGNC:110	Up-regula	RNA-seq	Human	HT-1080 c	Screened	Further stu
CBS	Cystathior	HGNC:155	Up-regula	RNA-seq	Human	HT-1080 c	Screened	Further stu
ATF4	Activating	HGNC:786	Up-regula	RNA-seq	Human	HT-1080 c	Screened	Further stu
ZNF419	Zinc finger	HGNC:206	Up-regula	RNA-seq	Human	HT-1080 c	Screened	Further stu
KLHL24	Kelch like 1	HGNC:259	Up-regula	RNA-seq	Human	HT-1080 c	Screened	Further stu
TRIB3	Tribbles ps	HGNC:162	Up-regula	RNA-seq	Human	HT-1080 c	Screened	Further stu
ZFP69B	ZFP69 zinc	HGNC:280	Up-regula	RNA-seq	Human	HT-1080 c	Screened	Further stu
ATP6V1G2	ATPase H-	HGNC:862	Up-regula	RNA-seq	Human	HT-1080 c	Screened	Further stu
VEGFA	Vascular e	HGNC:126	Up-regula	RNA-seq	Human	HT-1080 c	Screened	Further stu
GDF15	Growth dif	HGNC:301	Up-regula	RNA-seq	Human	HT-1080 c	Screened	Further stu
TUBE1	Tubulin ep	HGNC:207	Up-regula	RNA-seq	Human	HT-1080 c	Screened	Further stu
ARRDC3	Arrestin do	HGNC:292	Up-regula	RNA-seq	Human	HT-1080 c	Screened	Further stu
CEBPG	CCAAT en	HGNC:183	Up-regula	RNA-seq	Human	HT-1080 c	Screened	Further stu
SNORA16A	Small nucl	HGNC:326	Down-reg	RNA-seq	Human	HT-1080 c	Screened	Further stu
RGS4	Regulator	HGNC:100	Down-reg	RNA-seq	Human	HT-1080 c	Screened	Further stu
BLOC1S5-	BLOC1S5-	HGNC:420	Down-reg	RNA-seq	Human	HT-1080 c	Screened	Further stu
LOC39070	_NA_	_NA_	Down-reg	RNA-seq	Human	HT-1080 c	Screened	Further stu
EIF2S1	Eukaryotic	HGNC:326	Phosphory	Western b	Human	HT-1080 c	Deduced	_NA_
KIM-1	Kidney inju	_NA_	Down-reg	Real-time	Mice	Fer-1–trea	Deduced	_NA_
IL6	Interleukin	HGNC:601	Down-reg	Real-time	Mice	Fer-1–trea	Deduced	_NA_
CXCL2	C-X-C mo	HGNC:460	Down-reg	Real-time	Mice	Fer-1–trea	Deduced	_NA_
RELA	RELA prot	HGNC:995	Down-reg	Real-time	Mice	Fer-1–trea	Deduced	_NA_
HSD17B11	Hydroxyst	HGNC:229	Enriched in	Gene trap	Human	Chronic m	Screened	_NA_
AGPAT3	1-acylglyc	HGNC:326	Enriched in	Gene trap	Human	Chronic m	Screened	_NA_
SETD1B	SET doma	HGNC:291	Enriched in	Gene trap	Human	Chronic m	Screened	_NA_
HMOX1	Heme oxy	HGNC:501	Its express	Western b	Human	Pancreatic	Deduced	_NA_
TF	Transferrin	HGNC:117	Its express	Gene expr	Human	Patient PD	Deduced	_NA_
FTL	Ferritin lig	HGNC:399	Its express	Gene expr	Human	Patient PD	Deduced	_NA_
RPL8	Ribosomal	HGNC:103	Significant	Gene expr	Human	Patient PD	Deduced	_NA_

ATP5MC3	ATP synth	HGNC:843	Significant	Gene expr	Human	Patient PD	Deduced	_NA_
TFRC	Transferrir	HGNC:117	Expressior	Gene expr	Human	Patient PD	Deduced	_NA_
MAFG	MAF bZIP	HGNC:678	The intera	Western b	Human, m	HepG2, H	Deduced	_NA_
IL33	Interleukin	HGNC:160	IL-33 upre	Western b	Mice	C57BL/6 n	Deduced	_NA_
FTH1	Ferritin he	HGNC:397	An increas	Immunobl	Human	HT1080 ce	Validated	An error n
SLC40A1	Solute carr	HGNC:109	Erastin-inc	Q-PCR	Mice	Bone marr	Validated	Used as a
TF	Transferrir	HGNC:117	Erastin-inc	Q-PCR	Mice	Bone marr	Validated	Used as a
TFRC	Transferrir	HGNC:117	Erastin-inc	Q-PCR	Mice	Bone marr	Validated	Used as a
FTH1	Ferritin he	HGNC:397	Erastin-inc	Q-PCR, we	Mice	Bone marr	Validated	Used as a
GPX4	Glutathion	HGNC:455	Erastin-inc	Q-PCR, we	Mice	Bone marr	Validated	Used as a
HAMP	Hepcidin a	HGNC:155	Erastin-inc	Q-PCR	Mice	Bone marr	Deduced	_NA_
HSPB1	Heat shocl	HGNC:524	Erastin-inc	Q-PCR	Mice	Bone marr	Validated	Used as a
NFE2L2	Nuclear fa	HGNC:778	Erastin-inc	Q-PCR	Mice	Bone marr	Validated	Used as a
STEAP3	STEAP3 m	HGNC:245	Erastin-inc	Q-PCR	Mice	Bone marr	Deduced	_NA_
DRD5	Dopamine	HGNC:302	Ferroptoti	Western b	Human	PANC1 ce	Deduced	_NA_
GPX4	Glutathion	HGNC:455	Erastin prc	Western b	Human	PANC1 ce	Deduced	Has been
DRD4	Dopamine	HGNC:302	Antiferrop	Western b	Human	PANC1 ce	Deduced	_NA_
MAP3K5	Mitogen-a	HGNC:685	Cold stress	LDH assay	Human	A549 cells	Deduced	_NA_
MAPK14	Mitogen-a	HGNC:687	Cold stress	LDH assay	Human	A549 cells	Deduced	_NA_
SLC2A1	Solute carr	HGNC:110	Increased	ChIP analy	Human	H358, A54	Deduced	_NA_
SLC2A3	Solute carr	HGNC:110	Decreased	RT-qPCR	Human	H358, A54	Deduced	_NA_
SLC2A6	Solute carr	HGNC:110	Increased	RT-qPCR	Human	H358, A54	Deduced	_NA_
SLC2A8	Solute carr	HGNC:138	Decreased	RT-qPCR	Human	H358, A54	Deduced	_NA_
SLC2A12	Solute carr	HGNC:180	Increased	RT-qPCR	Human	H358, A54	Deduced	_NA_
GLUT13	_NA_	_NA_	Increased	RT-qPCR	Human	H358, A54	Deduced	_NA_
SLC2A14	Solute carr	HGNC:183	Decreased	RT-qPCR	Human	H358, A54	Deduced	_NA_
EIF2AK4	Eukaryotic	HGNC:196	CHAC1 de	Western b	Human	MDA-MB-	Deduced	_NA_
EIF2S1	Eukaryotic	HGNC:326	CHAC1 de	Western b	Human	MDA-MB-	Deduced	_NA_
ATF4	Activating	HGNC:786	CHAC1 de	Western b	Human	MDA-MB-	Deduced	_NA_
ALOX5	Arachidon	HGNC:435	Overexpre	Western b	Human	HEK293 ce	Deduced	Not essen
ALOX12	Arachidon	HGNC:429	Overexpre	Western b	Human	HEK293 ce	Deduced	Not essen
ALOX15	Arachidon	HGNC:433	Overexpre	Western b	Human	HEK293 ce	Deduced	Not essen
ALOX5	Arachidon	HGNC:435	Necessary	qRT-PCR,	Mice	C57BL/6 a	Deduced	_NA_
ACSF2	Acyl-CoA	HGNC:261	Increased	Western b	Rat	Wistar rats	Deduced	_NA_
IREB2	Iron respo	HGNC:611	Increased	Western b	Rat	Wistar rats	Deduced	_NA_
GPX4	Glutathion	HGNC:455	Upregulat	Western b	Rat	Wistar rats	Validated	Inhibits fer
HMGB1	High mobi	HGNC:498	Associatec	ELISA, wes	Human, m	HT1080 ar	Deduced	_NA_
HMOX1	Heme oxy	HGNC:501	Required f	RNA-seq,	Mice	Mice	Deduced	_NA_
NFE2L2	Nuclear fa	HGNC:778	DOX treat	RNA-seq,	Mice	Mice	Deduced	_NA_
ELAVL1	ELAV like	HGNC:331	Binds to a	RNA pulld	Human	H358, PC9	Deduced	_NA_
SLC3A2	Solute carr	HGNC:110	Strongly c	Western b	Human	HT-1080 c	Deduced	_NA_
SLC7A11	Solute carr	HGNC:110	Strongly c	Western b	Human	HT-1080 c	Deduced	_NA_
TFAP2C	Transcripti	HGNC:117	Activated	Behavioral	Mice	C57BL/6 n	Deduced	_NA_

SP1	Sp1 transc	HGNC:112	Activated	Behavioral	Mice	C57BL/6 n	Deduced	_NA_
HBA1	Hemoglot	HGNC:482	Upregulat	Tandem m	Human	HepG2, Be	Deduced	_NA_
NNMT	Nicotinam	HGNC:786	Upregulat	Tandem m	Human	HepG2, Be	Deduced	_NA_
PLIN4	Perilipin 4	HGNC:293	Upregulat	Tandem m	Human	HepG2, Be	Deduced	_NA_
HIC1	HIC ZBTB	HGNC:490	Essential fc	Immunohi	Human	HepG2, Be	Deduced	_NA_
STMN1	Stathmin 1	HGNC:651	Downregu	Tandem m	Human	HepG2, Be	Deduced	_NA_
RRM2	Ribonucle	HGNC:104	Downregu	Tandem m	Human	HepG2, Be	Deduced	_NA_
CAPG	Capping a	HGNC:147	Downregu	Tandem m	Human	HepG2, Be	Deduced	_NA_
HNF4A	Hepatocyt	HGNC:502	Essential fc	Immunohi	Human	HepG2, Be	Deduced	_NA_
NGB	Neuroglot	HGNC:140	Human ne	Cell death	Human	SH-SY5Y c	Deduced	_NA_
YWHAE	Tyrosine 3	HGNC:128	Required t	Mass spec	Human	HEK293T c	Deduced	_NA_
GABPB1	GA bindin	HGNC:407	Downregu	RT-qPCR,	Human	HepG2, Hu	Deduced	_NA_
AURKA	Aurora kin	HGNC:113	Inhibition	qRT-PCR,	Human	OE33, STK	Predicted	_NA_
MIR4715	microRNA	HGNC:416	Inhibition	qRT-PCR,	Human	OE33, STK	Predicted	_NA_
RIPK1	Receptor i	HGNC:100	Reduced e	Cell viabili	Human	Patient-de	Deduced	_NA_
PRDX1	Peroxiredc	HGNC:935	Necessary	Western b	Human	Primary cc	Deduced	_NA_
MIR30B	microRNA	HGNC:316	Upregulati	GSH assay	Human, ra	Trophobla	Deduced	_NA_
SLC7A11	Solute carr	HGNC:110	Silencing c	RT-qPCR,	Human	HT-1080 c	SLC7A11 :	Validated
GPX4	Glutathion	HGNC:455	RNAi-mec	Affinity-ba	Human, m	HRAS mut	GPX4 :-: Li	Validated
AKR1C1	Aldo-keto	HGNC:384	Up-regula	RNA-seq,	Human	DU-145 p	AKR1C1 :-	Validated
AKR1C2	Aldo-keto	HGNC:385	Up-regula	RNA-seq,	Human	DU-145 p	AKR1C2 :-	Validated
AKR1C3	Aldo-keto	HGNC:386	Up-regula	RNA-seq,	Human	DU-145 p	AKR1C3 :-	Validated
GPX4	Glutathion	HGNC:455	Knockout	Cell viabili	Human, m	Mouse em	GPX4 :-: Li	Validated
RB1	RB transcr	HGNC:988	Rb knock-	RNA interf	Human, m	HCC cell li	RB1 :-: Fer	Validated
HSPB1	Heat shocl	HGNC:524	Knockdow	RNAi, RT-	Human, m	HeLa and	HSPB1 :-:	Validated
HSF1	Heat shocl	HGNC:522	Knockdow	RNAi, RT-	Human, m	HeLa and	HSF1 :-: Fe	Validated
SLC7A11	Solute carr	HGNC:110	Overexpre	Western b	Human, m	H1299 cell	SLC7A11 :	Validated
GPX4	Glutathion	HGNC:455	Ex vivo, Gp	Viability as	Mice	T cells	GPX4 :-: F	Validated
GCLC	Glutamate	HGNC:431	RNAi knoc	RNAi, qPC	Mice	Mouse em	GCLC :+: C	Validated
SLC7A11	Solute carr	HGNC:110	Overexpre	Cell death	Human	H1299 cell	SLC7A11 :	Validated
NFE2L2	Nuclear fa	HGNC:778	NRF2 play	Cell Viabili	Human, m	HepG2, He	NFE2L2 :+	Validated
SQSTM1	Sequestos	HGNC:112	The intera	Immunopr	Human, m	HepG2, He	SQSTM1 :-	Validated
NQO1	NAD(P)H c	HGNC:287	Knockdow	qRT-PCR,	Human, m	HepG2, He	NQO1 :-: I	Validated
HMOX1	Heme oxy	HGNC:501	Knockdow	qRT-PCR,	Human, m	HepG2, He	HMOX1 :-	Validated
FTH1	Ferritin he	HGNC:397	Knockdow	qRT-PCR,	Human, m	HepG2, He	FTH1 :-: Fe	Validated
MUC1	Mucin 1, c	HGNC:750	MUC1-C (	shRNA, GS	Human	MDA-MB-	MUC1 :+:	Validated
SLC3A2	Solute carr	HGNC:110	Required f	Cell surviv	Mice	Mouse ES-	SLC3A2 :+	Validated
MT1G	Metallothi	HGNC:739	A negative	RNA interf	Human, m	HCC cells	MT1G :-: F	Validated
NFE2L2	Nuclear fa	HGNC:778	Required f	shRNA, qF	Human	Huh7 and	NFE2L2 :+	Deduced
SLC40A1	Solute carr	HGNC:109	Involved ir	Western b	Human	MDA MB 2	SLC40A1 :	Validated
SLC7A11	Solute carr	HGNC:110	Knockdow	siRNA, wes	Human	MDA MB 2	SLC7A11 :	Validated
GPX4	Glutathion	HGNC:455	Knockdow	siRNA, wes	Human	MDA MB 2	GPX4 :-: F	Validated
SLC7A11	Solute carr	HGNC:110	Inhibition	Cell viabili	Human, m	AMC-HN3	SLC7A11 :	Validated

CISD1	CDGSH irc	HGNC:308	Genetic in	Cytotoxicit	Human	HepG2 an	CISD1 :-:	Validated
SLC7A11	Solute carr	HGNC:110	Elevated le	Western b	Human	H1299 cell	SLC7A11 :	Validated
FANCD2	FA comple	HGNC:358	Inhibits era	Cell viabili	Mice	Bone marr	FANCD2 :-	Validated
GPX4	Glutathion	HGNC:455	Protects lip	RT-PCR, w	Human	Umbilical v	GPX4 :-: L	Validated
NFE2L2	Nuclear fa	HGNC:778	Nrf2 activa	Cell viabili	Mice	Head and	NFE2L2 :-:	Validated
FTMT	Ferritin mi	HGNC:173	Overexpre	Cell viabili	Mice, fly	SH-SY5Y c	FTMT :-: L	Validated
HSPA5	Heat shocl	HGNC:523	Negatively	Cell viabili	Human, m	Pancreatic	HSPA5 :+:	Validated
ATF4	Activating	HGNC:786	Inhibition	CCK8 cell	Human	Pancreatic	ATF4 :+: H	Validated
SLC7A11	Solute carr	HGNC:110	Slc7a11 de	Cell viabili	Mice	Mice, hepa	SLC7A11 :	Validated
GPX4	Glutathion	HGNC:455	Ferroptosi	RT-qPCR,	Mice	Forebrain	GPX4 :-: F	Validated
GPX4	Glutathion	HGNC:455	Downregu	Immunobl	Human	Human ma	GPX4 :-: L	Validated
HMOX1	Heme oxy	HGNC:501	Demonsra	RT-PCR, w	Mice	Renal prox	HMOX1 :-	Validated
ATF4	Activating	HGNC:786	ATF4 expr	siRNA, cell	Human	U87 and L	ATF4 :+: x	Validated
NFE2L2	Nuclear fa	HGNC:778	Nrf2 over	Cell growt	Human, ra	F98, U87 g	NFE2L2 :+	Validated
TP53	Tumor pro	HGNC:119	Inhibits fer	RNAi, live-	Human, m	HCT116, a	TP53 :+: N	Validated
SLC7A11	Solute carr	HGNC:110	Knockdow	shRNA, ce	Human	HCT116	SLC7A11 :	Validated
HELLS	Helicase, ly	HGNC:486	LSH inhibi	Plate-colo	Human, m	Lung canc	HELLS :+:	Validated
SCD	Stearoyl-C	HGNC:105	Depletion	shRNA, RT	Human	A549 canc	SCD :-: Fe	Validated
FADS2	Fatty acid	HGNC:357	Depletion	shRNA, RT	Human	A549 canc	FADS2 :-:	Validated
SRC	SRC proto	HGNC:112	Src-STAT3	Immunobl	Human	MCF-10A	SRC :+: ST	Validated
STAT3	Signal tran	HGNC:113	Src-STAT3	Immunobl	Human	MCF-10A	STAT3 :-:	Validated
NFE2L2	Nuclear fa	HGNC:778	ARF-medi	Gene trans	Human, m	H1299, Sa	NFE2L2 :+	Validated
PML	Promyeloc	HGNC:911	PML expre	Cell death	Human	IMR90 cell	PML :-: Fe	Validated
MTOR	Mechanist	HGNC:394	Necessary	Cell viabili	Mice	Cardiomyo	MTOR :-: I	Validated
NFS1	NFS1 cyste	HGNC:159	Suppressio	RNAi, cell	Human	MDA-MB-	NFS1 :-: T	Validated
TP63	Tumor pro	HGNC:159	Delta Np6	siRNA, cell	Human	ME-180 ce	TP63 :-: Fe	Validated
SLC7A11	Solute carr	HGNC:110	Overexper	Cell viabili	Human	MDA-MB-	SLC7A11 :	Validated
TP53	Tumor pro	HGNC:119	p53 stabili	Immunobl	Human	HT-1080 c	TP53 :+: C	Validated
CDKN1A	Cyclin dep	HGNC:178	Required t	CRISPR, sh	Human	HT-1080 c	CDKN1A :	Validated
MIR137	microRNA	HGNC:315	Suppresse	Immunobl	Human, m	Melanoma	MIR137 :-	Validated
SLC40A1	Solute carr	HGNC:109	Overexpre	Cell viabili	Mice	TM4 Serto	SLC40A1 :	Validated
GPX4	Glutathion	HGNC:455	Activation	Cell viabili	Mice	TM4 Serto	GPX4 :-: L	Validated
GPX4	Glutathion	HGNC:455	GPX4-ove	Immunohi	Human	LCL-K cell	GPX4 :-: 8	Deduced
ENPP2	Ectonucle	HGNC:335	Overexpre	Cell viabili	Rat	H9c2 cells	ENPP2 :-:	Validated
VDAC2	Voltage de	HGNC:126	Overexpre	Western b	Human	HT1080 ce	VDAC2 :-:	Validated
FH	Fumarate	HGNC:370	FH inactiva	CRISPR/Ca	Human	UOK262, H	FH :+: GPX	Validated
CISD2	CDGSH irc	HGNC:242	Overexpre	Cell viabili	Human	Head and	CISD2 :-: L	Validated
SLC40A1	Solute carr	HGNC:109	A negative	Cell viabili	Human	SH-SY5Y c	SLC40A1 :	Validated
MIR9-1	microRNA	HGNC:316	Overexpre	Immunobl	Human	A375 and	MIR9 :-: G	Validated
MIR9-2	microRNA	HGNC:316	Overexpre	Immunobl	Human	A375 and	MIR9 :-: G	Validated
MIR9-3	microRNA	HGNC:316	Overexpre	Immunobl	Human	A375 and	MIR9 :-: G	Validated
CBS	Cystathior	HGNC:155	Inhibition	Cell viabili	Human, m	HepG2 cel	CBS :-: Fer	Validated
NFE2L2	Nuclear fa	HGNC:778	Associated	Cell viabili	Human, m	HNC cell li	NFE2L2 :+	Validated

SQSTM1	Sequestos	HGNC:112	Inhibition	siRNA, qP	Human	HN3 cells	SQSTM1 :-	Validated
GPX4	Glutathion	HGNC:455	Overexpre	Cell viabili	Human	HCT116, L	GPX4 :-: F	Validated
ISCU	Iron-sulfu	HGNC:298	Over expre	Cell viabili	Human	HL60, KG1	ISCU :+: G	Validated
FTH1	Ferritin he	HGNC:397	FTH recon	Cell viabili	Human	HL60, KG1	FTH1 :-: Li	Validated
ACSL3	Acyl-CoA	HGNC:357	Required f	Dead cell	Human	HEK293, H	ACSL3 :-: I	Validated
OTUB1	OTU deub	HGNC:230	Inactivatio	CRISPR/Ca	Human, m	H1299, SK	OTUB1 :+:	Validated
CD44	CD44 mol	HGNC:168	Knockdow	Western b	Human	H1299 cell	CD44 :+: S	Validated
LINC0033	Long inter	HGNC:338	Overexpre	qRT-PCR,	Human	A549 and	(LINC0033	Validated
STAT3	Signal trar	HGNC:113	Upregulat	Cell viabili	Human	Osteosarc	STAT3 :-: I	Validated
BRD4	Bromodor	HGNC:135	Inhibition	Cell prolife	Human	MDA-MB-	BRD4 :-: F	Validated
PRDX6	Peroxired	HGNC:167	A negative	Western b	Human	H1299 cell	PRDX6 :-:	Validated
MIR17	microRNA	HGNC:315	Protects e	Lentivirus	Human	HUVEC ce	MIR17 :-:	Validated
SCD	Stearoyl-C	HGNC:105	Inhibition	Cell viabili	Human, m	OVCAR-4,	SCD :+: Co	Validated
SESN2	Sestrin 2	HGNC:207	Has cytop	MTT assay	Human, m	HepG2 an	SESN2 :-: I	Validated
NF2	Neurofibr	HGNC:777	Genetic in	Cell death	Human, m	Mouse em	NF2 :-: YA	Validated
ARNTL	Aryl hydro	HGNC:701	Degration	Cytotoxicit	Human, m	Calu-1, TH	ARNTL :-:	Validated
HIF1A	Hypoxia in	HGNC:491	Destabilizi	Cytotoxicit	Human, m	Calu-1, TH	HIF1A :-:	Validated
JUN	Jun proto-	HGNC:620	O-GlcNAc	Immunoflu	Human, m	Bel-7402,	JUN :+: GS	Deduced
CA9	Carbonic a	HGNC:138	Inhibition	qRT-PCR,	Human	Mesotheli	CA9 :-: Fe	Validated
HSPA5	Heat shoc	HGNC:523	Knockdow	Fluorescer	Human, m	Pancreatic	GRP78 :-:	Validated
TMBIM4	Transmem	HGNC:242	Protects a	Cell viabili	Human, m	Hep G2, H	S1R :-: Fer	Validated
HSPA5	Heat shoc	HGNC:523	Serves as	Cell viabili	Human, m	Glioma cel	HSPA5 :+:	Predicted
PLIN2	Perilipin 2	HGNC:248	An indispe	Cell prolife	Human, m	SGC7901	PLIN2 :-: F	Screened
MIR212	microRNA	HGNC:315	Overexpre	Controlled	Mice	HT22 and	(miR-212-	Validated
Fer1HCH	Ferritin 1 H	_NA_	Reduced h	RT-qPCR,	Drosophil	Fly	Fer1HCH :	Predicted
AIFM2	Apoptosis	HGNC:214	A glutathi	Expression	Human, m	MCF-7, H	FPS1 :+: C	Validated
AIFM2	Apoptosis	HGNC:214	A potent f	Synthetic I	Human, m	U-2 OS ce	FPS1 :+: C	Validated
LAMP2	Lysosomal	HGNC:650	Knockdow	Cell viabili	Human	ARPE-19,	LAMP2 :+:	Validated
ZFP36	ZFP36 ring	HGNC:128	ZFP36 pla	Cell viabili	Human, m	Hepatic st	ZFP36 :-:	Validated
GPX4	Glutathion	HGNC:455	Depletion	Quantitati	Human, m	H9c2 and	GPX4 :-: Li	Validated
PROM2	Prominin 2	HGNC:206	Induced b	RNA-seq,	Human	MCF10A, I	Prominin2	Validated
CHMP5	Charged n	HGNC:269	Ferroptosi	RNAi, wes	Human, m	PANC1 an	CHMP5 :-	Validated
CHMP6	Charged n	HGNC:256	Ferroptosi	RNAi, wes	Human, m	PANC1 an	CHMP6 :-	Validated
AKR1C1	Aldo-keto	HGNC:384	Inhibition	RNAi, cell	Human	MeWo, A2	AKR1C1 :-	Validated
AKR1C2	Aldo-keto	HGNC:385	Inhibition	RNAi, cell	Human	MeWo, A2	AKR1C2 :-	Validated
AKR1C3	Aldo-keto	HGNC:386	Inhibition	RNAi, cell	Human	MeWo, A2	AKR1C3 :-	Validated
CBS	Cystathior	HGNC:155	Knockdow	RNAi, cell	Human	SKOV3 an	CBS :+: Cy	Validated
NFE2L2	Nuclear fa	HGNC:778	Geneticall	RNAi, cell	Human	SKOV3 an	NFE2L2 :+	Validated
CAV1	Caveolin 1	HGNC:152	Cav-1 defi	Immunoflu	Human, m	LO2 cells,	CAV-1 :+:	Validated
GCH1	GTP cyclol	HGNC:419	Gch1 over	CRISPR ac	Human	HT-1080,7	GCH1 :+: I	Validated

org/ferrdb/)

Caution	Protein en	UniProtKB	PMID	Remark
_NA_	60S ribosc	P62917 (R	22632970	_NA_
_NA_	Iron-respc	P48200 (IF	22632970	_NA_
_NA_	ATP synth	P48201 (A	22632970	ATP5G3 in article
_NA_	Citrate syr	O75390 (C	22632970	_NA_
_NA_	ER membr	Q15006 (E	22632970	TTC35 in article
_NA_	Medium-c	Q96CM8 (	22632970	_NA_
The preser	NADPH o	Q9Y5S8 (N	22632970	_NA_
The preser	Cytochron	P04839 (C	22632970	NOX2 in article
The preser	NADPH o	Q9HBY0 (I	22632970	_NA_
The preser	NADPH o	Q9NPH5 (	22632970	_NA_
The preser	NADPH o	Q96PH1 (I	22632970	_NA_
The preser	Dual oxida	Q9NRD9 (	22632970	_NA_
The preser	Dual oxida	Q9NRD8 (	22632970	_NA_
_NA_	Glucose-6	P11413 (G	22632970	_NA_
_NA_	6-phosph	P52209 (6	22632970	_NA_
_NA_	Voltage-d	P45880 (V	22632970	_NA_
Also involv	Phosphati	P42336 (P	24739485	PI3Kalpha in article
Also involv	Receptor-	P36888 (FI	24739485	_NA_
Transient (	Non-spec	P22307 (N	25402683	_NA_
The acetyl	Cellular tu	P04637 (P	25799988	_NA_
Author pre	Long-chai	O60488 (A	25965523	_NA_
Author pre	Lysophosp	Q6P1A2 (N	25965523	_NA_
Inferred as	GTPase NI	P01111 (R	26157704	_NA_
Inferred as	GTPase KF	P01116 (R	26157704	_NA_
Inferred as	GTPase HI	P01112 (R	26157704	_NA_
_NA_	Serotransf	P02787 (T	26166707	Transferrin in article
_NA_	Transferrir	P02786 (T	26166707	Tfr in article
_NA_	Transferrir	Q9UP52 (T	26166707	Tfr in article
_NA_	Sodium-c	Q9H2H9 (	26166707	_NA_
_NA_	Neutral an	Q15758 (A	26166707	_NA_
_NA_	Glutamina	Q9UI32 (G	26166707	_NA_
_NA_	Aspartate	P17174 (A	26166707	_NA_
_NA_	Cysteine--	P49589 (S	26184909	CARS in article
_NA_	Cellular tu	P04637 (P	26218928	p53 in article
_NA_	Arachidon	P09917 (L	26235588	5-LOX in article
_NA_	Kelch-like	Q14145 (K	26403645	_NA_
Other artic	Heme oxy	P09601 (H	26405158	HO-1 in article
_NA_	Cellular tu	P04637 (P	26943586	p53 in article
_NA_	Cellular tu	P04637 (P	27034505	p53 in article
_NA_	Glutamina	Q9UI32 (G	27034505	_NA_
_NA_	Autophag	Q9H1Y0 (A	27245739	_NA_

_NA_	Ubiquitin-	O95352 (A	27245739	_NA_
_NA_	Nuclear re	Q13772 (N	27245739	_NA_
_NA_	Serotransf	P02787 (T	27441659	Transferrin in article
_NA_	Arachidon	P09917 (L	27506793	_NA_
_NA_	Arachidon	P18054 (L	27506793	_NA_
_NA_	Arachidon	O75342 (L	27506793	_NA_
_NA_	Arachidon	P16050 (L	27506793	_NA_
_NA_	Arachidon	O15296 (L	27506793	_NA_
_NA_	Hydroperc	Q9BYJ1 (L	27506793	_NA_
_NA_	Phosphory	P15735 (P	27506793	_NA_
_NA_	Transferrir	P02786 (T	27514700	_NA_
_NA_	Cytoplasm	P21399 (A	27514700	_NA_
_NA_	Iron-respc	P48200 (IF	27514700	_NA_
_NA_	Sodium-cc	Q9H2H9 (	27514700	_NA_
_NA_	Glutamina	Q9UI32 (G	27514700	_NA_
_NA_	_NA_	_NA_	27514700	Not found in HGNC
_NA_	Serine/thr	O75385 (U	27514700	_NA_
_NA_	Ubiquitin-	Q9NT62 (A	27514700	_NA_
_NA_	Cysteine p	Q86TL0 (A	27514700	_NA_
_NA_	Autophag	Q9H1Y0 (A	27514700	_NA_
_NA_	Beclin-1	Q14457 (E	27514700	_NA_
_NA_	Microtubu	Q9H492 (N	27514700	_NA_
_NA_	Gamma-a	P60520 (G	27514700	_NA_
_NA_	Gamma-a	Q9H0R8 (C	27514700	_NA_
_NA_	Autophag	Q676U5 (A	27514700	_NA_
_NA_	WD repea	Q5MNZ9 (	27514700	_NA_
_NA_	WD repea	Q9Y4P8 (V	27514700	_NA_
_NA_	Sorting ne	O95219 (S	27514700	_NA_
_NA_	Autophag	O75143 (A	27514700	_NA_
_NA_	Serine/thr	Q8IYT8 (U	27514700	_NA_
_NA_	Nuclear re	Q13772 (N	27514700	_NA_
_NA_	Long-chai	O60488 (A	27565726	_NA_
_NA_	Cellular tu	P04637 (P	27705786	_NA_
Activated	Diamine a	P21673 (S	27698118	_NA_
_NA_	Arachidon	P16050 (L	27698118	_NA_
_NA_	Long-chai	O60488 (A	27842066	_NA_
_NA_	Lysophosp	Q6P1A2 (N	27842066	_NA_
_NA_	Arachidon	P16050 (L	27842066	15-LOX in article
_NA_	Long-chai	O60488 (A	27842070	_NA_
_NA_	Kelch-like	Q14145 (K	28012440	_NA_
_NA_	Epidermal	P00533 (E	28297659	_NA_
_NA_	NADPH ox	Q9NPH5 (	28297659	_NA_
_NA_	Mitogen-a	P27361 (M	28297659	ERK1 in article

_NA_	Mitogen-a	P28482 (M	28297659	ERK2 in article
_NA_	BH3-intera	P55957 (B	28384611	_NA_
_NA_	Long-chai	O60488 (A	28551825	_NA_
_NA_	Zinc finger	P37275 (Z	28678785	_NA_
_NA_	Kelch-like	Q14145 (K	28805788	_NA_
_NA_	Dipeptidyl	P27487 (D	28813679	_NA_
_NA_	Arachidon	P16050 (L	28837253	_NA_
_NA_	Arachidon	P18054 (L	28837253	_NA_
_NA_	Cyclin-dep	P42771 (C	28985506	ARF in article
_NA_	Phosphati	P30086 (P	29053969	_NA_
_NA_	Suppresso	O15524 (S	29081404	_NA_
_NA_	Cysteine d	Q16878 (C	29144989	_NA_
_NA_	Transcripti	P10242 (M	29144989	C-Myb in article
_NA_	Heme oxy	P09601 (H	29274359	HO-1 in article
_NA_	Mitogen-a	P45983 (M	29330409	JNK1 in article
_NA_	Mitogen-a	P45984 (M	29330409	JNK2 in article
_NA_	Mitogen-a	P28482 (M	29330409	ERK2 in article
_NA_	Mitogen-a	P27361 (M	29330409	ERK1 in article
_NA_	Neutral an	Q15758 (A	29348676	_NA_
_NA_	Glutathion	Q9BUX1 (	29383104	_NA_
_NA_	Mitogen-a	Q16539 (M	29436589	p38 MAPK in article
Also trigge	Putative u	Q9H8W2 (	29588351	P53RRA in article
_NA_	NADPH ox	Q9NPH5 (	29702192	_NA_
_NA_	Aspartate	P17174 (A	30035324	_NA_
_NA_	Beclin-1	Q14457 (B	30057310	_NA_
_NA_	5'-AMP-a	P54646 (A	30057310	PRKAA in article
_NA_	5'-AMP-a	Q13131 (A	30057310	AMPK alpha in article
_NA_	ELAV-like	Q15717 (E	30081711	_NA_
_NA_	Ubiquitin	Q92560 (B	30202049	_NA_
_NA_	Cellular tu	P04637 (P	30321484	P53 in article
_NA_	Multidrug	P33527 (M	30726737	MRP1 in article
_NA_	Long-chai	O60488 (A	30737476	_NA_
_NA_	_NA_	_NA_	30787392	_NA_
_NA_	Activin rec	P36896 (A	30804470	ALK4 in article
_NA_	TGF-beta	P36897 (T	30804470	ALK5 in article
_NA_	Ubiquitin	Q92560 (B	30907299	_NA_
_NA_	Endothelia	Q99814 (E	30962421	HIF-2alpha in article
_NA_	Hypoxia-in	Q9Y5L2 (H	30962421	_NA_
_NA_	Hypoxia-in	Q16665 (H	30962421	HIF-1alpha in article
_NA_	Arachidon	P18054 (L	30962574	_NA_
_NA_	Long-chai	O60488 (A	30962574	_NA_
_NA_	Heme oxy	P09601 (H	31036877	_NA_
_NA_	Interferon	P01579 (IF	31043744	IFNgamma in article

_NA_	Anoctamir	Q4KMQ2 (	31060306	_NA_
_NA_	Phosphati	Q14693 (L	31061954	_NA_
_NA_	High mobi	P09429 (H	31105999	_NA_
_NA_	Tumor ne	P21580 (T	31160087	Zinc lipoprotein A20 in article
_NA_	Toll-like re	O00206 (T	31196626	_NA_
_NA_	NADPH o	Q9NPH5 (	31196626	_NA_
_NA_	Cyclic AMI	P18847 (A	31273299	_NA_
_NA_	Serine-pro	Q13315 (A	31320750	_NA_
_NA_	YY1-assoc	Q9H869 (Y	31341276	YAP in article
_NA_	Egl nine h	Q96KS0 (E	31355331	_NA_
_NA_	Inositol ox	Q9UGB7 (	31437128	_NA_
_NA_	Tafazzin	Q16635 (T	31484063	_NA_
_NA_	Protein LY	Q86UE4 (L	31527591	_NA_
_NA_	Isocitrate (	O75874 (I	31591388	_NA_
_NA_	NAD-dep	Q96EB6 (S	31610175	_NA_
_NA_	Tafazzin	Q16635 (T	31641008	_NA_
_NA_	Beclin-1	Q14457 (E	31650158	Beclin1 in article
_NA_	F-box/WD	Q969H0 (F	31679460	_NA_
_NA_	Pannexin-	Q96RD7 (I	31694915	_NA_
_NA_	DnaJ hom	O75190 (D	31701262	_NA_
_NA_	Transcripti	O14867 (E	31740582	_NA_
_NA_	Long-chai	O60488 (A	31789401	_NA_
_NA_	Lon protea	P36776 (L	31822343	_NA_
Prostaglar	P35354 (P	24439385	May promote ferroptosis.	
Dual speci	P28562 (D	24439385	May promote ferroptosis.	
Nitric oxid	P35228 (N	24439385	May promote ferroptosis.	
Neutrophi	P19878 (N	24439385	May promote ferroptosis.	
Metallothi	P25713 (M	24439385	May promote ferroptosis.	
Polyubiqui	P0CG48 (U	24439385	May promote ferroptosis.	
Serum alb	P02768 (A	24439385	May promote ferroptosis.	
Thioredox	Q16881 (T	24439385	May promote ferroptosis.	
Sulfiredoxi	Q9BYN0 (S	24439385	May promote ferroptosis.	
Glutathion	P18283 (G	24439385	May promote ferroptosis.	
BCL2/ader	Q12983 (E	24439385	May promote ferroptosis.	
Serine/thr	O95747 (C	24439385	May promote ferroptosis.	
Selenopro	Q9BQE4 (S	24439385	SELS in article. May promote ferroptosis.	
Angiopoie	O43827 (A	24439385	May promote ferroptosis.	
Glutathion	Q9BUX1 (C	24844246	_NA_	
Cystine/gl	Q9UPY5 (X	24844246	May inhibit ferroptosis.	
DNA dama	Q9NX09 (I	24844246	May promote ferroptosis.	
_NA_	_NA_	24844246	Not found in HGNC. May promote ferroptosis.	
Asparagin	P08243 (A	24844246	May promote ferroptosis.	
TSC22 dor	Q99576 (T	24844246	May promote ferroptosis.	

DDIT3 ups	P0DPQ6 (I	24844246	May promote ferroptosis.
Jun dimeri	Q8WYK2 (	24844246	May promote ferroptosis.
Sestrin-2	P58004 (SI	24844246	May promote ferroptosis.
Neutral an	P43007 (S,	24844246	May promote ferroptosis.
Phosphoe	Q16822 (F	24844246	May promote ferroptosis.
Thioredox	Q9H3M7 (	24844246	May promote ferroptosis.
Very low-(	P98155 (V	24844246	May promote ferroptosis.
Alanine an	Q8TD30 (/	24844246	May promote ferroptosis.
Phosphos	Q9Y617 (S	24844246	May promote ferroptosis.
Leucine ric	Q8IV03 (LI	24844246	C9ORF150 in article. May promote ferroptosis.
Large neu	Q01650 (L	24844246	May promote ferroptosis.
Homocyst	Q15011 (H	24844246	May promote ferroptosis.
X-box-bin	P17861 (X	24844246	May promote ferroptosis.
Cyclic AMI	P18847 (A	24844246	May promote ferroptosis.
4F2 cell-su	P08195 (4	24844246	May promote ferroptosis.
Cystathior	P35520 (C	24844246	May promote ferroptosis.
Cyclic AMI	P18848 (A	24844246	May promote ferroptosis.
Zinc finger	Q96HQ0 (	24844246	May promote ferroptosis.
Kelch-like	Q6TFL4 (K	24844246	May promote ferroptosis.
Tribbles h	Q96RU7 (T	24844246	May promote ferroptosis.
Zinc finger	Q9UJL9 (Z	24844246	ZNF643 in article. May promote ferroptosis.
V-type pr	O95670 (V	24844246	May promote ferroptosis.
Vascular e	P15692 (V	24844246	May promote ferroptosis.
Growth/di	Q99988 (C	24844246	May promote ferroptosis.
Tubulin ep	Q9UJT0 (T	24844246	May promote ferroptosis.
Arrestin d	Q96B67 (A	24844246	May promote ferroptosis.
CCAAT/en	P53567 (C	24844246	May promote ferroptosis.
_NA_	_NA_	24844246	May inhibit ferroptosis.
Regulator	P49798 (R	24844246	May inhibit ferroptosis.
_NA_	_NA_	24844246	MUTED-TXNDC5 in article. May inhibit ferroptosis.
_NA_	_NA_	24844246	Not found in HGNC. May inhibit ferroptosis.
Eukaryotic	P05198 (IF	24844246	eIF2alpha in article. May promote ferroptosis.
_NA_	_NA_	25385600	Not found in HGNC. May promote ferroptosis.
Interleukin	P05231 (IL	25385600	IL-6 in article. May promote ferroptosis.
C-X-C mc	P19875 (C	25385600	May promote ferroptosis.
Transcripti	Q04206 (T	25385600	p65 in article. May promote ferroptosis.
Estradiol 1	Q8NBQ5 (	25965523	May promote ferroptosis.
1-acyl-sn-	Q9NRZ7 (I	25965523	May promote ferroptosis.
Histone-ly	Q9UPS6 (S	25965523	May promote ferroptosis.
Heme oxy	P09601 (H	26097885	HO-1 in article. May promote ferroptosis.
Serotransf	P02787 (T	26097885	May promote ferroptosis.
Ferritin lig	P02792 (FI	26097885	May promote ferroptosis.
60S ribosc	P62917 (R	26097885	May promote ferroptosis.

ATP synth	P48201 (A	26097885	ATP5G3 in article. May promote ferroptosis.
Transferrir	P02786 (T	26097885	May inhibit ferroptosis.
Transcripti	O15525 (N	26403645	May inhibit ferroptosis.
Interleukin	O95760 (II	27352622	IL-33 in article. May promote ferroptosis.
Ferritin he	P02794 (FI	27514700	_NA_
Solute cari	Q9NP59 (S	27773819	May promote ferroptosis.
Serotransf	P02787 (T	27773819	May promote ferroptosis.
Transferrir	P02786 (T	27773819	May promote ferroptosis.
Ferritin he	P02794 (FI	27773819	May inhibit ferroptosis.
Phospholi	P36969 (G	27773819	May inhibit ferroptosis.
Hepcidin	P81172 (H	27773819	May inhibit ferroptosis.
Heat shoc	P04792 (H	27773819	May inhibit ferroptosis.
Nuclear fa	Q16236 (N	27773819	NRF2 in article. May inhibit ferroptosis.
Metallorec	Q658P3 (S	27773819	May inhibit ferroptosis.
D(1B) dop	P21918 (D	27793671	May promote ferroptosis.
Phospholi	P36969 (G	27793671	May inhibit ferroptosis.
D(4) dopa	P21917 (D	27793671	May inhibit ferroptosis.
Mitogen-a	Q99683 (N	28887319	ASK1 in article. May promote ferroptosis.
Mitogen-a	Q16539 (N	28887319	p38 in article. May promote ferroptosis.
Solute cari	P11166 (G	28900510	GLUT1 in article. May inhibit ferroptosis.
Solute cari	P11169 (G	28900510	GLUT3 in article. May inhibit ferroptosis.
Solute cari	Q9UGQ3 (	28900511	GLUT6 in article. May inhibit ferroptosis.
Solute cari	Q9NY64 ((	28900512	GLUT8 in article. May inhibit ferroptosis.
Solute cari	Q8TD20 ((	28900513	GLUT12 in article. May inhibit ferroptosis.
_NA_	_NA_	28900514	Not found in HGNC. May inhibit ferroptosis.
Solute cari	Q8TDB8 ((	28900515	GLUT14 in article. May inhibit ferroptosis.
eIF-2-alpl	Q9P2K8 (E	29383104	GCN2 in article. May promote ferroptosis.
Eukaryotic	P05198 (IF	29383104	eIF2alpha in article. May promote ferroptosis.
Cyclic AMI	P18848 (A	29383104	May promote ferroptosis.
Arachidon	P09917 (L(	29632885	5-LOX in article
Arachidon	P18054 (L(	29632885	p12-LOX in article
Arachidon	P16050 (L(	29632885	15-LOX-1 in article
Arachidon	P09917 (L(	30294906	May promote ferroptosis.
Medium-c	Q96CM8 (	30539824	There is a corrigendum for this article. May promote ferroptosis
Iron-respc	P48200 (IF	30539824	There is a corrigendum for this article. May promote ferroptosis
Phospholi	P36969 (G	30539824	There is a corrigendum for this article.
High mobi	P09429 (H	30686534	May promote ferroptosis.
Heme oxy	P09601 (H	30692261	May promote ferroptosis.
Nuclear fa	Q16236 (N	30692261	NRF2 in article.
ELAV-like	Q15717 (E	30787392	May inhibit ferroptosis.
4F2 cell-su	P08195 (4	31043744	May inhibit ferroptosis.
Cystine/gl	Q9UPY5 ()	31043744	May inhibit ferroptosis.
Transcripti	Q92754 (A	31056284	May inhibit ferroptosis.

Transcripti	P08047 (SI	31056284	May inhibit ferroptosis.	
Hemoglob	P69905 (H	31108460	May promote ferroptosis.	
Nicotinam	P40261 (N	31108460	May promote ferroptosis.	
Perilipin-4	Q96Q06 (I	31108460	May promote ferroptosis.	
Hypermetl	Q14526 (H	31108460	May promote ferroptosis.	
Stathmin	P16949 (S	31108460	May inhibit ferroptosis.	
Ribonucle	P31350 (R	31108460	May inhibit ferroptosis.	
Macropha	P40121 (C	31108460	May inhibit ferroptosis.	
Hepatocyt	P41235 (H	31108460	May inhibit ferroptosis.	
Neuroglob	Q9NPG2 (	31405213	May inhibit ferroptosis.	
14-3-3 pr	P62258 (1	31581313	14-3-3epsilon in article. May promote ferroptosis.	
GA-bindir	Q06547 (C	31700067	May inhibit ferroptosis.	
Aurora kin	O14965 (A	31740746	May inhibit ferroptosis.	
_NA_	_NA_	31740746	miR-4715-3p in article. May promote ferroptosis.	
Receptor-	Q13546 (R	31827280	May promote ferroptosis.	
Peroxiredo	Q06830 (P	31901729	May promote ferroptosis.	
_NA_	_NA_	31926626	miR-30b-5p in article. May promote ferroptosis.	
_NA_	Cystine/gl	Q9UPY5 (X	22632970	_NA_
_NA_	Phospholi	P36969 (G	24439385	_NA_
_NA_	Aldo-keto	Q04828 (A	24844246	_NA_
_NA_	Aldo-keto	P52895 (A	24844246	_NA_
_NA_	Aldo-keto	P42330 (A	24844246	_NA_
_NA_	Phospholi	P36969 (G	25402683	_NA_
_NA_	Retinoblas	P06400 (R	25444922	_NA_
_NA_	Heat shocl	P04792 (H	25728673	_NA_
_NA_	Heat shocl	Q00613 (H	25728673	_NA_
_NA_	Cystine/gl	Q9UPY5 (X	25799988	_NA_
_NA_	Phospholi	P36969 (G	25824823	_NA_
_NA_	Glutamate	P48506 (G	26166707	_NA_
_NA_	Cystine/gl	Q9UPY5 (X	26218928	_NA_
_NA_	Nuclear fa	Q16236 (N	26403645	NRF2 in article
_NA_	Sequestos	Q13501 (S	26403645	p62 in article.
_NA_	NAD(P)H (	P15559 (N	26403645	_NA_
Article (PM	Heme oxy	P09601 (H	26403645	HO1 in article
_NA_	Ferritin he	P02794 (FI	26403645	_NA_
_NA_	Mucin-1	P15941 (M	26930718	_NA_
_NA_	4F2 cell-su	P08195 (4	26945935	CD98hc in article
_NA_	Metallothi	P13640 (M	27015352	MT-1G in article
Its ability t	Nuclear fa	Q16236 (N	27015352	NRF2 in article
_NA_	Solute carr	Q9NP59 (S	27441659	Ferroportin-1 (FPN) in article
_NA_	Cystine/gl	Q9UPY5 (X	27441659	_NA_
_NA_	Phospholi	P36969 (G	27441659	_NA_
_NA_	Cystine/gl	Q9UPY5 (X	27477897	_NA_

_NA_	CDGSH irc	Q9NZ45 ((	27510639	_NA_
_NA_	Cystine/gl	Q9UPY5 (>	27705786	_NA_
_NA_	Fanconi ar	Q9BXW9 (	27773819	_NA_
_NA_	Phospholi	P36969 (G	27964880	_NA_
_NA_	Nuclear fa	Q16236 (N	28012440	NRF2 in article
_NA_	Ferritin	Q8N4E7 (F	28066232	_NA_
_NA_	Endoplasn	P11021 (B	28130223	_NA_
_NA_	Cyclic AMI	P18848 (A	28130223	_NA_
_NA_	Cystine/gl	Q9UPY5 (>	28195347	_NA_
_NA_	Phospholi	P36969 (G	28212525	_NA_
_NA_	Phospholi	P36969 (G	28297659	_NA_
Article (PM	Heme oxy	P09601 (H	28515173	HO-1 in article
_NA_	Cyclic AMI	P18848 (A	28553953	_NA_
_NA_	Nuclear fa	Q16236 (N	28805788	Nrf2 in article
Promotes	Cellular tu	P04637 (P	28813679	_NA_
_NA_	Cystine/gl	Q9UPY5 (>	28813679	_NA_
_NA_	Lymphoid	Q9NRZ9 (I	28900510	LSH in article
_NA_	Acyl-CoA	O00767 (A	28900510	SCD1 in article
_NA_	Acyl-CoA	O95864 (F	28900510	_NA_
_NA_	Proto-onc	P12931 (SI	28972104	_NA_
_NA_	Signal trar	P40763 (S	28972104	_NA_
_NA_	Nuclear fa	Q16236 (N	28985506	NRF2 in article
_NA_	Protein PN	P29590 (P	29081404	_NA_
_NA_	Serine/thr	P42345 (N	29127238	_NA_
_NA_	Cysteine d	Q9Y697 (N	29168506	_NA_
_NA_	Tumor pro	Q9H3D4 (	29212036	Delta Np63 alpha in article
_NA_	Cystine/gl	Q9UPY5 (>	29274359	_NA_
Found to p	Cellular tu	P04637 (P	29346757	p53 in article
_NA_	Cyclin-dep	P38936 (C	29346757	Encoding p21
_NA_	_NA_	_NA_	29348676	_NA_
_NA_	Solute car	Q9NP59 (S	29436589	Fpn in article
_NA_	Phospholi	P36969 (G	29436589	_NA_
The autho	Phospholi	P36969 (G	29463878	_NA_
_NA_	Ectonucle	Q13822 (E	29551679	_NA_
_NA_	Voltage-d	P45880 (V	29569437	_NA_
_NA_	Fumarate	P07954 (FI	29917289	_NA_
_NA_	CDGSH irc	Q8N5K1 ((	29928961	_NA_
_NA_	Solute car	Q9NP59 (S	29949159	Ferroportin in article
_NA_	_NA_	_NA_	30035324	miR-9 in article
_NA_	_NA_	_NA_	30035324	miR-9 in article
_NA_	_NA_	_NA_	30035324	miR-9 in article
_NA_	Cystathior	P35520 (C	30258181	_NA_
_NA_	Nuclear fa	Q16236 (N	30339884	Nrf2 in article

Not test c	Sequestos	Q13501 (S	30339884	p62 in article.
_NA_	Phospholi	P36969 (G	30524291	_NA_
_NA_	Iron-sulfu	Q9H1K1 (I	30557609	_NA_
_NA_	Ferritin he	P02794 (FI	30557609	FTH in article
_NA_	Long-chai	O95573 (A	30686757	_NA_
_NA_	Ubiquitin t	Q96FW1 (	30709928	_NA_
_NA_	CD44 anti	P16070 (C	30709928	_NA_
_NA_	Putative u	Q6ZUF6 (N	30787392	_NA_
_NA_	Signal trar	P40763 (S	30811078	_NA_
_NA_	Bromodor	O60885 (E	30988278	_NA_
_NA_	Peroxiredc	P30041 (P	31036877	_NA_
_NA_	_NA_	_NA_	31160087	miR-17-92 in article
_NA_	Acyl-CoA	O00767 (A	31270077	SCD1 in article
_NA_	Sestrin-2	P58004 (SI	31323261	Sesn2 in article
_NA_	Merlin	P35240 (M	31341276	Also known as merlin
_NA_	Aryl hydro	O00327 (E	31355331	_NA_
_NA_	Hypoxia-ir	Q16665 (H	31355331	_NA_
_NA_	Transcripti	P05412 (JU	31394193	c-Jun in article
_NA_	Carbonic a	Q16790 (C	31442913	_NA_
_NA_	Endoplasn	P11021 (B	31456633	GRP78 in article
_NA_	Protein life	Q9HC24 (I	31507082	S1R in article
_NA_	Endoplasn	P11021 (B	31519193	_NA_
_NA_	Perilipin-2	Q99541 (F	31520166	Also known as ADRP
_NA_	_NA_	_NA_	31533781	miR-212-5p in article
_NA_	Ferritin	H1UUD2 (	31568497	_NA_
_NA_	Apoptosis	Q9BRQ8 (	31634899	FSP1 in article
_NA_	Apoptosis	Q9BRQ8 (	31634900	FSP1 in article
_NA_	Lysosome	P13473 (L	31672277	_NA_
_NA_	mRNA dec	P26651 (T	31679460	_NA_
_NA_	Phospholi	P36969 (G	31685805	_NA_
_NA_	Prominin-	Q8N271 (I	31735663	Prominin2 in article
_NA_	Charged n	Q9NZZ3 (	31761326	_NA_
_NA_	Charged n	Q96FZ7 (C	31761326	_NA_
_NA_	Aldo-keto	Q04828 (A	31780644	_NA_
_NA_	Aldo-keto	P52895 (A	31780644	_NA_
_NA_	Aldo-keto	P42330 (A	31780644	_NA_
_NA_	Cystathior	P35520 (C	31819185	_NA_
_NA_	Nuclear fa	Q16236 (N	31819185	NRF2 in article
_NA_	Caveolin-1	Q03135 (C	31877357	Cav-1 in article
_NA_	GTP cyclol	P30793 (G	31989025	_NA_

S.
S.
