# Supplementary material for: A Novel Prognostic Ferroptosis-Related lncRNA Signature Associated with Immune Landscape in Invasive Breast Cancer
Source: Dis Markers. 2022 Mar 20;2022:9168556. doi: 10.1155/2022/9168556 (PMC8961446; doi:10.1155/2022/9168556)
Supplement: Supplementary 7 — Table S2: ferroptosis-related lncRNAs were differentially expressed. [file 9168556.f7.pdf]

Table S2:ferroptosis-related lncRNA were differentially expressed

gene	conMean	treatMean	logFC	pValue	fdr
LINC02716	0.82256	0.257646	-1.67473	1.28E-27	9.55E-27
MYOSLID	0.569478	0.277352	-1.03792	3.72E-21	1.72E-20
CYTOR	2.238146	4.711003	1.07373	3.32E-27	2.37E-26
CDKN2B-AS	0.059682	0.253717	2.087841	2.58E-38	4.14E-37
TPT1-AS1	2.827375	0.963909	-1.5525	1.51E-43	3.65E-42
LINC00924	0.81831	0.164801	-2.31192	4.98E-53	2.80E-51
LINC02202	1.490435	0.184053	-3.01754	5.22E-62	1.61E-59
LMNTD2-AS	0.693413	2.103086	1.600722	4.83E-10	1.04E-09
AC121247.	0.582099	0.186998	-1.63824	5.42E-39	9.57E-38
AC099684.	0.107475	0.227436	1.081454	0.027263	0.0317
AC005544.	0.128018	0.399742	1.642723	7.26E-08	1.36E-07
A2M-AS1	1.411118	0.590681	-1.25639	1.11E-46	3.82E-45
AC106782.	0.755304	1.682357	1.155355	3.02E-17	1.04E-16
LINC01569	0.948716	1.983779	1.064203	3.34E-21	1.55E-20
DLEU2	0.399191	0.923729	1.21039	5.33E-31	4.88E-30
AC233992.	0.080095	0.307111	1.938984	1.22E-22	6.33E-22
AC093110.	2.652573	0.532947	-2.31533	1.55E-52	8.33E-51
U62317.1	0.492388	3.70504	2.911622	1.11E-30	9.83E-30
AC107214.	0.291234	0.61107	1.069157	1.46E-17	5.10E-17
MIR200CHG	5.130717	11.2867	1.137391	1.90E-16	6.22E-16
AC144450.	0.437776	1.077341	1.299209	3.68E-09	7.53E-09
AL049539.	0.070788	0.270813	1.935726	3.59E-09	7.37E-09
AC245884.	0.11018	0.261314	1.245919	0.008088	0.009928
MIR99AHG	2.214714	0.618339	-1.84065	2.46E-60	4.34E-58
AL139246.	0.442893	1.671502	1.916114	5.08E-24	2.96E-23
AC006946.	0.789403	0.351008	-1.16926	4.56E-25	2.85E-24
AF131215.	2.253314	0.84233	-1.41959	1.85E-36	2.44E-35
AF015262.	0.01701	0.251573	3.886535	9.81E-19	3.81E-18
LINC00641	3.517679	1.368358	-1.36218	5.59E-49	2.16E-47
MAL2-AS1	0.059316	0.228208	1.943853	5.96E-12	1.46E-11
ZNF350-AS	0.361745	1.42026	1.973109	0.000252	0.000358
GAS1RR	0.992551	0.210338	-2.23843	5.99E-56	5.70E-54
AC067817.	0.569548	0.211319	-1.43039	5.48E-33	5.69E-32
AP005131.	0.191987	0.825567	2.104378	3.03E-23	1.69E-22
AC004585.	0.234566	0.861445	1.876766	2.29E-28	1.76E-27
LINC02100	0.130213	0.335767	1.366594	1.73E-05	2.74E-05
AC092171.	0.268966	0.740906	1.461866	1.76E-27	1.30E-26
AC064799.	0.052352	0.627296	3.582823	0.014368	0.017254
AC105137.	0.092957	0.259634	1.481851	3.15E-23	1.74E-22
FARSA-AS1	0.061676	0.219731	1.832965	7.62E-06	1.24E-05
LINC00922	0.024513	0.55973	4.513117	1.31E-53	8.07E-52
EMX2OS	1.11975	0.251162	-2.15649	1.01E-42	2.26E-41
AL365436.	0.098224	0.448575	2.191209	1.09E-17	3.83E-17
AC037198.	1.046288	4.018546	1.941394	3.13E-11	7.39E-11
UBE2Q1-AS	0.174783	0.381724	1.126964	2.05E-15	6.25E-15
AL356215.	0.184969	0.409422	1.146308	0.000729	0.001003
LINC01235	5.674947	2.014265	-1.49435	2.30E-31	2.19E-30
YTHDF3-AS	0.902779	1.866695	1.048041	6.14E-13	1.62E-12
AC245014.	0.295585	0.651164	1.139449	0.000446	0.000621

LINC01705	0.023258	1.209578	5.700618	7.66E-56	6.31E-54
LAMC1-AS1	1.111548	0.375609	-1.56527	1.04E-40	2.01E-39
SOX9-AS1	1.454946	0.560023	-1.37741	1.09E-26	7.26E-26
AL451050.	0.126064	0.345931	1.456332	7.93E-20	3.35E-19
AL133230.	0.095638	0.270025	1.497438	1.44E-05	2.29E-05
EMSLR	1.228518	3.280109	1.416825	4.98E-16	1.59E-15
AC062004.	0.888567	0.270231	-1.71729	7.58E-36	9.37E-35
LINC01614	0.202066	8.924098	5.46481	8.16E-63	1.01E-59
AC105219.	0.051195	0.65021	3.666818	1.43E-33	1.52E-32
AC020663.	0.221412	0.876876	1.985638	6.04E-36	7.61E-35
AC132192.	0.513891	1.105042	1.104566	2.64E-21	1.24E-20
AC124312.	1.38052	0.667115	-1.0492	7.98E-27	5.33E-26
AC024075.	4.602156	2.16298	-1.08929	1.39E-29	1.15E-28
AC009133.	0.72252	1.575613	1.124803	3.49E-45	1.00E-43
AC007285.	0.107437	0.26214	1.286847	1.22E-12	3.17E-12
LINC01341	0.209657	0.461144	1.137187	1.04E-05	1.68E-05
ETV7-AS1	0.15211	0.334567	1.137178	6.74E-06	1.10E-05
AL359878.	0.10569	0.227628	1.106835	0.000131	0.000192
AL158212.	2.180127	1.028133	-1.08438	3.06E-47	1.11E-45
AC027228.	0.137949	0.379486	1.45991	1.02E-05	1.66E-05
Z99289.1	0.505971	0.169716	-1.57593	5.69E-35	6.51E-34
AL357992.	0.14784	0.577264	1.965196	2.55E-22	1.28E-21
AP005131.	0.055011	0.336106	2.611126	0.032061	0.037139
LINC00702	0.98487	0.354943	-1.47235	4.43E-24	2.61E-23
AP003419.	0.407349	1.186337	1.542177	6.12E-14	1.73E-13
VLDLR-AS1	0.576883	0.163216	-1.8215	1.53E-36	2.03E-35
MIR4435-2	1.341948	2.730588	1.024883	1.23E-33	1.34E-32
LINC02446	0.346742	0.932399	1.427083	0.014392	0.017254
LINC02544	0.448953	5.346031	3.573833	5.29E-49	2.11E-47
AC010331.	0.110254	0.459049	2.05782	1.09E-28	8.50E-28
AC103769.	0.077359	0.251174	1.69904	0.009568	0.011663
LYPLAL1-A	1.732947	0.471908	-1.87665	7.17E-39	1.23E-37
BCL2L1-AS	0.139857	0.537517	1.94236	0.033208	0.038432
CD2BP2-DT	1.780501	5.476307	1.62092	2.74E-31	2.59E-30
MYB-AS1	0.126549	0.334953	1.404265	1.86E-05	2.94E-05
CADM3-AS1	0.834681	0.141942	-2.55592	6.48E-56	5.72E-54
AC006270.	0.064566	0.406234	2.653465	0.000505	0.0007
AC011465.	0.137868	0.398977	1.53302	1.17E-07	2.16E-07
AL023803.	0.030924	0.340528	3.460954	1.77E-45	5.22E-44
AC006946.	1.045166	0.514786	-1.02169	4.23E-23	2.32E-22
AL121829.	0.078383	0.312553	1.995481	6.58E-19	2.60E-18
AC010503.	8.008529	18.15559	1.180805	7.02E-34	7.75E-33
LINC02273	0.117999	0.249339	1.07933	0.037649	0.043287
AC084125.	0.180082	0.54559	1.599164	9.01E-22	4.40E-21
MIR100HG	5.43745	1.764481	-1.62369	5.83E-56	5.70E-54
LINC01943	0.129221	0.495628	1.939419	2.40E-39	4.36E-38
AC004816.	0.458542	0.201157	-1.18873	6.62E-28	4.99E-27
PPP1R14B-	0.611005	2.127835	1.800131	3.44E-35	4.05E-34
C1orf220	0.206485	0.478959	1.213869	3.08E-15	9.31E-15
TRHDE-AS1	2.899746	0.079112	-5.19588	3.12E-55	2.15E-53
CARMN	2.066636	0.286558	-2.85039	2.27E-58	2.81E-56

NRIR	0.185912	0.375294	1.013404	0.002723	0.003529
AC007938.	2.514325	0.859747	-1.54819	1.02E-40	1.99E-39
BRWD1-AS1	0.094673	0.232094	1.293685	1.04E-06	1.79E-06
AC093535.	0.868234	0.239019	-1.86096	1.00E-32	1.01E-31
MIR205HG	19.90148	7.755454	-1.35959	4.14E-21	1.90E-20
AC004884.	0.104928	0.38084	1.859784	7.64E-06	1.24E-05
SOCAR	0.047571	0.264087	2.472851	2.31E-37	3.44E-36
AC108134.	1.334625	0.468678	-1.50977	2.65E-44	6.68E-43
RBMS3-AS3	0.989074	0.168985	-2.54918	9.70E-59	1.33E-56
DNAH10OS	0.469638	0.971433	1.048566	6.30E-11	1.46E-10
AC011447.	0.145229	0.302858	1.060311	1.62E-11	3.86E-11
AP003555.	1.844274	0.351297	-2.39229	1.22E-32	1.21E-31
MIAT	0.269291	0.753724	1.484873	1.07E-18	4.13E-18
LINC00639	0.651441	0.253618	-1.36098	3.68E-45	1.03E-43
AL359715.	0.529194	0.258905	-1.03138	1.37E-29	1.13E-28
LINC00511	0.112847	0.79407	2.814903	1.29E-30	1.13E-29
USP30-AS1	0.430129	1.095811	1.34916	1.16E-08	2.30E-08
AC105020.	0.731902	0.328667	-1.15502	2.15E-39	3.97E-38
AL590133.	0.128757	0.372382	1.532129	4.06E-19	1.63E-18
LINC01929	0.055203	0.66318	3.586576	6.14E-39	1.07E-37
PCAT6	2.379168	7.198252	1.597189	1.58E-35	1.90E-34
SRRM2-AS1	0.218401	0.481317	1.140009	2.77E-19	1.13E-18
AP005131.	0.032939	0.304503	3.208587	0.039128	0.044904
AL590226.	0.942092	0.314469	-1.58295	6.47E-40	1.23E-38
AC010326.	4.749327	11.10406	1.225293	4.65E-20	1.98E-19
AC134312.	0.088424	0.844005	3.254733	3.36E-50	1.54E-48
LINC01857	0.392164	1.11476	1.507203	1.74E-23	9.95E-23
AC096921.	1.533181	0.331087	-2.21125	2.62E-59	4.04E-57
AP001528.	6.732928	0.861631	-2.96609	4.05E-56	4.55E-54
AC083880.	0.346472	0.912015	1.39632	4.60E-22	2.26E-21
AC091057.	0.216326	0.643815	1.573438	3.58E-36	4.57E-35
AC009171.	0.401345	0.914375	1.187943	4.64E-16	1.49E-15
AC138904.	0.11091	0.629532	2.504893	1.40E-37	2.14E-36
AC040169.	0.192147	0.411208	1.097659	8.60E-12	2.10E-11
MAPT-IT1	0.081418	0.381833	2.229522	0.000791	0.001081
LINC02195	0.058091	0.320369	2.463341	7.04E-17	2.38E-16
LINC00092	0.583868	0.223841	-1.38316	3.22E-41	6.63E-40
RHPN1-AS1	0.88277	2.872387	1.70214	6.67E-45	1.79E-43
AC011676.	0.13382	0.300376	1.16648	3.76E-07	6.64E-07
AC022509.	1.729073	0.824539	-1.06834	8.50E-28	6.37E-27
AC022196.	0.017885	0.572772	5.00114	7.02E-11	1.62E-10
U62317.2	2.530317	5.211081	1.042265	1.12E-24	6.73E-24
AL445423.	0.855767	0.281786	-1.60262	7.68E-35	8.71E-34
AC120498.	1.248652	5.986779	2.261408	1.69E-11	4.02E-11
AC004988.	0.080942	0.225895	1.4807	4.95E-25	3.08E-24
NR4A1AS	2.324953	0.859259	-1.43604	2.01E-28	1.55E-27
AC092119.	0.323817	0.697904	1.107849	3.31E-16	1.08E-15
AL136084.	2.322535	1.004593	-1.20909	3.05E-31	2.85E-30
AC048341.	0.803213	0.383594	-1.0662	1.91E-30	1.65E-29
LINC01352	0.879113	0.262927	-1.74139	2.02E-39	3.77E-38
Z97989.1	1.147018	0.389827	-1.55698	2.47E-53	1.46E-51

AL591686.	1.980022	0.087775	-4.49556	2.79E-62	1.15E-59
SCAT2	0.273305	0.656476	1.264231	7.92E-19	3.09E-18
AL117329.	0.027215	0.302556	3.474719	7.60E-18	2.69E-17
AC012073.	0.374688	1.113791	1.571718	4.15E-37	5.97E-36
AC020765.	0.432664	0.867202	1.00312	4.04E-10	8.80E-10
AL109741.	1.665044	0.45212	-1.88078	5.72E-46	1.81E-44
JARID2-AS	0.123648	0.266269	1.106645	7.23E-06	1.18E-05
MAFG-DT	1.517914	4.691141	1.627849	3.18E-41	6.63E-40
AL031846.	0.654342	0.323488	-1.01633	4.69E-27	3.27E-26
AL683807.	0.162575	0.502437	1.627837	2.69E-20	1.18E-19
AC083843.	2.281715	0.982894	-1.21501	3.46E-27	2.46E-26
AC023794.	0.822187	0.389788	-1.07678	1.43E-25	9.05E-25
AC024451.	0.071682	0.241576	1.752799	1.43E-10	3.22E-10
AL691482.	0.68765	1.474286	1.10027	0.000175	0.000254
AC079298.	0.723433	0.261759	-1.46662	3.08E-29	2.45E-28
AL121672.	0.1924	0.403674	1.069081	0.002716	0.003522
AP001189.	1.756538	0.870544	-1.01275	3.73E-20	1.62E-19
AC027698.	0.042328	0.262292	2.631501	0.00199	0.002614
AC124319.	0.16686	0.785423	2.234835	2.16E-25	1.36E-24
AL049838.	1.798179	0.814702	-1.14219	1.42E-38	2.35E-37
AC137630.	0.16842	0.353744	1.070647	1.47E-07	2.67E-07
AP005131.	0.33336	1.342833	2.010126	1.14E-24	6.82E-24
C9orf106	0.093205	0.293363	1.654211	1.37E-11	3.29E-11
LRRC8C-DT	0.775683	0.281926	-1.46015	4.68E-50	2.07E-48
AL157838.	0.31253	0.860841	1.46175	1.01E-18	3.91E-18
AC025175.	0.999132	0.489286	-1.03	3.91E-35	4.52E-34
TYMSOS	0.314181	1.352308	2.105758	6.59E-37	9.22E-36
LINC01589	1.074876	0.204131	-2.39661	9.55E-55	6.22E-53
AC073130.	0.673057	0.189823	-1.82607	6.33E-47	2.24E-45
SIAH2-AS1	0.506665	1.305482	1.36548	5.03E-09	1.02E-08
AL445426.	1.996224	0.165209	-3.59491	2.66E-61	6.57E-59
MIR3150BH	0.035846	0.226971	2.66262	2.69E-27	1.95E-26
AC010300.	0.171882	0.357082	1.054832	0.014841	0.017775
KIAA1671-	4.042715	2.007705	-1.00978	4.18E-20	1.79E-19
AL022341.	0.350118	0.771244	1.139347	3.55E-12	8.90E-12
AC068473.	0.032891	0.233785	2.829427	2.69E-33	2.82E-32
AC007998.	1.061569	0.360206	-1.5593	5.99E-32	5.87E-31
MAGI2-AS3	3.963294	0.913597	-2.11707	2.01E-62	1.15E-59
AP003071.	0.92358	0.20074	-2.20191	2.55E-52	1.31E-50
HSD11B1-A	11.78852	0.664666	-4.14861	2.56E-51	1.26E-49
SNHG25	0.643237	2.2572	1.811112	2.24E-24	1.33E-23
AL163051.	0.264568	0.557834	1.076199	4.23E-22	2.09E-21
LINC02693	1.045174	0.494867	-1.07863	1.57E-46	5.26E-45
LINC00337	0.037201	0.250147	2.749379	2.30E-44	5.93E-43
AP005131.	0.112405	0.453606	2.012729	4.94E-08	9.46E-08
AP001010.	0.174472	0.362708	1.055814	2.16E-08	4.20E-08
LNCOG	0.841653	0.243851	-1.78723	7.19E-41	1.43E-39
AC069234.	0.583873	0.270422	-1.11044	1.92E-30	1.65E-29
LINC02587	4.114152	0.348347	-3.562	5.94E-61	1.22E-58
AC107031.	0.035419	0.259188	2.871398	8.69E-09	1.72E-08
GNG12-AS1	0.64799	0.199674	-1.69833	3.03E-55	2.15E-53

AL390294.	0.444126	1.836857	2.048199	1.44E-08	2.83E-08
AC087239.	0.153712	0.469927	1.612204	2.28E-18	8.56E-18
AP000941.	0.511482	0.22802	-1.16552	9.40E-31	8.42E-30
AL158847.	1.030535	0.35345	-1.54382	7.81E-17	2.62E-16
AC116025.	0.016812	0.238273	3.825074	9.81E-36	1.19E-34
AL136115.	0.154496	0.478734	1.63165	0.000946	0.00128
AP000695.	0.341632	0.807536	1.241084	3.33E-19	1.36E-18
LINC01354	0.854864	0.223375	-1.93623	6.49E-51	3.08E-49
AC022146.	0.189299	0.535937	1.501397	5.82E-08	1.10E-07
AL031985.	0.883029	1.922089	1.122142	4.25E-34	4.73E-33
AC092801.	0.028972	0.621986	4.424138	1.52E-13	4.18E-13
AC093278.	4.463566	1.538218	-1.53694	5.85E-45	1.61E-43
MIR210HG	0.55279	1.614969	1.546702	3.21E-21	1.50E-20
AC092718.	2.127149	6.036555	1.504804	1.06E-32	1.07E-31
LINC01711	0.345254	0.929091	1.428161	2.50E-20	1.12E-19
U73166.1	0.303303	0.642571	1.083094	6.73E-10	1.43E-09
LINC02657	0.064255	0.355389	2.46751	8.08E-32	7.86E-31
MIR924HG	0.228096	0.549718	1.269048	3.34E-12	8.41E-12
AC104667.	0.540956	1.581505	1.547714	1.91E-29	1.55E-28
AC004687.	0.388976	0.829356	1.092311	0.000768	0.001052
AC109322.	0.650874	1.680513	1.368452	1.60E-22	8.26E-22
LINC01655	0.059918	0.250631	2.064515	1.47E-13	4.08E-13
AC009093.	0.184104	0.754594	2.03518	6.55E-36	8.18E-35
LINC01215	0.117258	0.261117	1.155018	0.041639	0.047654
AF178030.	0.117953	2.045965	4.116497	2.05E-07	3.70E-07
MIR22HG	11.74398	4.137019	-1.50526	5.49E-43	1.26E-41
AC093423.	0.452915	0.203666	-1.15304	3.32E-39	5.95E-38
AC092484.	0.016412	0.250204	3.930267	1.48E-14	4.29E-14
AL162171.	1.041674	0.442038	-1.23666	2.93E-36	3.81E-35
LINC02257	0.069197	0.291961	2.077003	7.49E-18	2.67E-17
AC005730.	0.095476	0.275451	1.528584	1.22E-07	2.23E-07
AGAP1-IT1	0.168928	1.105305	2.709963	4.40E-37	6.25E-36
AL358075.	0.068647	0.275137	2.002894	2.42E-18	9.07E-18
AC008966.	0.650983	0.281791	-1.208	9.16E-38	1.43E-36
TFAP2A-AS	0.430215	0.966685	1.167987	1.84E-22	9.37E-22
AC083906.	0.210657	1.197915	2.507559	4.02E-10	8.78E-10
LINC01402	0.925346	0.235364	-1.9751	1.25E-42	2.76E-41
AC010336.	0.078937	0.221303	1.487244	4.81E-14	1.37E-13
AL139393.	1.275876	3.059804	1.261952	1.64E-20	7.38E-20
AC022211.	0.161771	0.397034	1.295313	5.08E-13	1.34E-12
LINC01094	0.665256	1.46641	1.140307	5.24E-27	3.64E-26
AC092142.	0.209562	0.564667	1.430025	1.53E-08	3.00E-08
AC008115.	0.949738	2.189105	1.204741	4.41E-12	1.10E-11
POLH-AS1	0.302067	0.782922	1.374002	1.07E-36	1.45E-35
AL136115.	0.179965	0.463563	1.36505	3.89E-06	6.47E-06
HOXA-AS2	1.057464	0.362364	-1.5451	3.12E-49	1.28E-47
AP000553.	0.159403	0.604548	1.923174	8.53E-26	5.46E-25
ACTA2-AS1	5.990954	0.98026	-2.61155	4.16E-42	9.02E-41
RNF157-AS	0.124982	0.290473	1.21669	7.62E-11	1.75E-10
AC090825.	0.73893	0.303544	-1.28353	1.64E-34	1.84E-33
SIDT1-AS1	0.124239	0.288044	1.213169	0.02042	0.024106

STPG3-AS1	0.192449	0.391805	1.02566	0.001856	0.002446
AC048382.	0.974691	0.350157	-1.47694	9.27E-34	1.01E-32
UBE2R2-AS	0.08573	0.220174	1.360769	0.000971	0.001313
AC012213.	0.128876	0.316445	1.295972	3.57E-27	2.52E-26
U47924.1	0.081288	0.306384	1.914232	1.70E-22	8.70E-22
AC013652.	0.054015	0.506044	3.227842	6.36E-27	4.32E-26
AC103706.	0.507255	1.802904	1.829539	1.20E-32	1.19E-31
AC098869.	0.156919	0.3309	1.076373	4.63E-07	8.08E-07
ZFHX4-AS1	0.466436	0.226411	-1.04274	4.36E-21	2.00E-20
MEG3	4.108826	1.487778	-1.46557	7.51E-44	1.86E-42
LINC01485	1.9682	0.152284	-3.69204	1.04E-45	3.22E-44
AC108134.	0.76447	2.058377	1.428975	1.06E-17	3.73E-17
AL139220.	0.098843	0.275176	1.477151	2.32E-13	6.32E-13
AP001434.	0.021326	0.488383	4.517322	8.19E-39	1.37E-37
DDX11-AS1	0.160107	0.426592	1.413821	4.19E-31	3.90E-30
AP000251.	0.166271	0.916884	2.463199	3.18E-37	4.68E-36
LINC01351	0.070726	0.553716	2.968844	0.001729	0.002288
LINC02528	0.024405	0.221371	3.181222	3.67E-19	1.48E-18
HCG11	8.27157	3.437112	-1.26696	1.48E-49	6.29E-48
THSD4-AS1	0.014133	0.308552	4.448342	0.0005	0.000694
AC105219.	0.259396	1.026413	1.984381	2.40E-38	3.90E-37
AP000695.	0.148534	0.410501	1.466596	3.12E-28	2.38E-27
TMPO-AS1	0.304905	0.998126	1.710863	9.32E-48	3.49E-46
MIR155HG	0.441706	0.938351	1.087041	2.51E-11	5.95E-11
LIPE-AS1	1.085159	0.469421	-1.20895	1.91E-33	2.02E-32
AL021368.	0.667604	0.248666	-1.42479	8.73E-45	2.30E-43
LINC01703	0.65649	1.417516	1.110521	3.40E-14	9.79E-14
SENCR	1.08391	0.439875	-1.30108	2.31E-35	2.74E-34
AC073195.	0.247414	0.563632	1.187824	2.16E-21	1.02E-20
LINC00665	2.956334	6.087296	1.041992	1.49E-31	1.44E-30
AC020916.	21.16723	6.680958	-1.66371	1.81E-37	2.72E-36
AL121832.	1.135383	2.645982	1.220624	9.97E-16	3.13E-15
HLA-F-AS1	0.788944	0.381088	-1.0498	1.31E-37	2.02E-36
LINC02580	0.874103	0.190186	-2.20039	1.11E-55	8.57E-54
RNF144A-A	0.14874	0.366624	1.301506	3.06E-17	1.05E-16
AC121757.	1.520269	0.67789	-1.1652	7.52E-19	2.94E-18
MMP2-AS1	0.149319	0.448845	1.587824	2.21E-16	7.21E-16
AL445228.	0.234793	0.484251	1.044368	2.88E-08	5.58E-08
AP001189.	0.8584	0.251667	-1.77013	9.58E-33	9.78E-32
AC138696.	0.747957	2.429774	1.699795	1.78E-29	1.45E-28
AP005131.	0.089224	0.781422	3.1306	4.62E-16	1.48E-15
AL157373.	0.11013	0.259078	1.23418	0.000732	0.001005
AC110792.	0.901747	0.439155	-1.03799	3.31E-29	2.63E-28
AC002546.	6.261363	0.233315	-4.74613	1.13E-45	3.39E-44
AC008764.	7.558064	3.615917	-1.06366	7.94E-36	9.72E-35
AL356311.	0.49733	3.959292	2.992968	1.37E-07	2.51E-07
