# Supplementary material for: A Novel Prognostic Ferroptosis-Related lncRNA Signature Associated with Immune Landscape in Invasive Breast Cancer
Source: Dis Markers. 2022 Mar 20;2022:9168556. doi: 10.1155/2022/9168556 (PMC8961446; doi:10.1155/2022/9168556)
Supplement: Supplementary 8 — Table S3: ten ferroptosis-related lncRNA risk model. [file 9168556.f8.pdf]

Table S3:ten ferroptosis-related lncRNAs risk model

id	coef	HR	HR.95L	HR.95H	pvalue
CYTOR	0.079688	1.08295	1.003118	1.169134	0.041385
`LMNTD2-A	-0.13324	0.875252	0.753406	1.016803	0.08149
AP005131.	-0.70568	0.493771	0.255518	0.954177	0.035771
`LYPLAL1-	0.103827	1.109408	1.008559	1.220342	0.032742
`USP30-AS	-0.29405	0.745239	0.583062	0.952524	0.018853
`RHPN1-AS	0.142674	1.153354	1.053591	1.262563	0.001995
AC004988.	0.720037	2.05451	1.186141	3.558609	0.010199
AC079298.	0.644338	1.904726	1.11389	3.257039	0.018572
`HSD11B1-	-0.22614	0.797608	0.594693	1.069758	0.131103
LINC01655	0.480779	1.617334	0.952468	2.746308	0.075126
