# Supplementary material for: A Novel Prognostic Ferroptosis-Related lncRNA Signature Associated with Immune Landscape in Invasive Breast Cancer
Source: Dis Markers. 2022 Mar 20;2022:9168556. doi: 10.1155/2022/9168556 (PMC8961446; doi:10.1155/2022/9168556)
Supplement: Supplementary 9 — Table S4: gene enrichment analysis for ferroptosis-related lncRNAs based on the TCGA-BRCA entire set. [file 9168556.f9.pdf]

Table S4:Gene enrichment analysis for ferroptosis-related lncRNAs based on TCGA-B

NAME	GS<br>fc	GS DETAIL	SIZE	ES	NES	NOM p-val	FDR q-val	FWER p-va
KEGG_ADIF	KEGG_ADIF	Details .	67	-0.56504	-2.11399	0	0.044548	0.033
KEGG_GLYC	KEGG_GLYC	Details .	77	-0.5317	-2.10386	0	0.026329	0.038
KEGG_PRIM	KEGG_PRIM	Details .	35	-0.85153	-2.0588	0	0.033204	0.059
KEGG_ARAC	KEGG_ARAC	Details .	58	-0.5616	-2.02064	0	0.036852	0.083
KEGG_T_CE	KEGG_T_CE	Details .	108	-0.57887	-2.01998	0.012048	0.029631	0.083
KEGG_INTE	KEGG_INTE	Details .	46	-0.77359	-2.0128	0.006369	0.026137	0.088
KEGG_ETHE	KEGG_ETHE	Details .	33	-0.57757	-2.01216	0.002058	0.022403	0.088
KEGG_AUTC	KEGG_AUTC	Details .	50	-0.7015	-1.97712	0.008114	0.029461	0.125
KEGG_FC_E	KEGG_FC_E	Details .	79	-0.50823	-1.97046	0.00611	0.028277	0.129
KEGG_ASTH	KEGG_ASTH	Details .	28	-0.74548	-1.94621	0.002119	0.032565	0.155
KEGG_HEMA	KEGG_HEMA	Details .	85	-0.59158	-1.88377	0.020964	0.049745	0.221
KEGG_TYPE	KEGG_TYPE	Details .	41	-0.73974	-1.88186	0.012579	0.046101	0.221
KEGG_VIRA	KEGG_VIRA	Details .	68	-0.59141	-1.87662	0.016227	0.043876	0.228
KEGG_ALLC	KEGG_ALLC	Details .	35	-0.80601	-1.85868	0.012146	0.047834	0.254
KEGG_CYTC	KEGG_CYTC	Details .	264	-0.48531	-1.8584	0.010799	0.044748	0.255
KEGG_ANTI	KEGG_ANTI	Details .	81	-0.59615	-1.8525	0.020325	0.04477	0.27
KEGG_ALPH	KEGG_ALPH	Details .	19	-0.60031	-1.8424	0.007737	0.045751	0.287
KEGG_GNRH	KEGG_GNRH	Details .	101	-0.43918	-1.81628	0	0.051646	0.311
KEGG_GRAF	KEGG_GRAF	Details .	37	-0.77215	-1.81575	0.028398	0.049228	0.312
KEGG_CHEM	KEGG_CHEM	Details .	188	-0.49177	-1.8131	0.025478	0.047751	0.317
KEGG_VEGF	KEGG_VEGF	Details .	76	-0.45896	-1.80355	0.010438	0.048795	0.335
KEGG_JAK_	KEGG_JAK_	Details .	155	-0.45833	-1.8024	0.013043	0.047109	0.337
KEGG_CYTC	KEGG_CYTC	Details .	55	-0.51211	-1.79347	0.014523	0.048155	0.352
KEGG_NOTC	KEGG_NOTC	Details .	47	-0.51599	-1.79341	0.010776	0.046148	0.352
KEGG_NATU	KEGG_NATU	Details .	132	-0.50862	-1.79237	0.022222	0.044565	0.352
KEGG_RIG_	KEGG_RIG_	Details .	71	-0.46903	-1.76593	0.016529	0.053151	0.389
KEGG_LEIS	KEGG_LEIS	Details .	70	-0.59511	-1.76273	0.039419	0.052761	0.398
KEGG_PHOS	KEGG_PHOS	Details .	76	-0.47315	-1.75927	0.01417	0.051911	0.4
KEGG_APOF	KEGG_APOF	Details .	87	-0.46278	-1.73207	0.025948	0.059642	0.43
KEGG_BASE	KEGG_BASE	Details .	35	-0.59352	-1.71252	0.041916	0.065694	0.462
KEGG_MAPK	KEGG_MAPK	Details .	267	-0.39392	-1.71163	0.010373	0.063823	0.464
KEGG_LINC	KEGG_LINC	Details .	29	-0.52323	-1.70466	0.013436	0.064769	0.475
KEGG_CELL	KEGG_CELL	Details .	131	-0.4995	-1.70062	0.068323	0.064191	0.479
KEGG_NOD_	KEGG_NOD_	Details .	62	-0.49562	-1.68617	0.054326	0.068388	0.497
KEGG_ABC_	KEGG_ABC_	Details .	44	-0.47744	-1.66096	0.034765	0.077479	0.539
KEGG_PPAR	KEGG_PPAR	Details .	69	-0.47962	-1.65123	0.010504	0.0794	0.552
KEGG_NON_	KEGG_NON_	Details .	54	-0.43897	-1.58778	0.028398	0.109856	0.628
KEGG_B_CE	KEGG_B_CE	Details .	75	-0.48463	-1.58521	0.079592	0.108404	0.631
KEGG_TOLL	KEGG_TOLL	Details .	102	-0.42291	-1.58223	0.053061	0.107329	0.637
KEGG_ACUT	KEGG_ACUT	Details .	57	-0.43025	-1.56099	0.042194	0.116639	0.66
KEGG_ENDC	KEGG_ENDC	Details .	181	-0.37754	-1.54387	0.043033	0.124462	0.679
KEGG_LONG	KEGG_LONG	Details .	70	-0.38816	-1.53428	0.022495	0.128737	0.696
KEGG_SNAF	KEGG_SNAF	Details .	38	-0.452	-1.53308	0.05501	0.126646	0.697
KEGG_FC_G	KEGG_FC_G	Details .	96	-0.40954	-1.5167	0.06746	0.13518	0.714
KEGG_GLYC	KEGG_GLYC	Details .	49	-0.39781	-1.50046	0.050813	0.143865	0.735
KEGG_TYPE	KEGG_TYPE	Details .	47	-0.39865	-1.47711	0.06237	0.156917	0.757
KEGG_NICC	KEGG_NICC	Details .	24	-0.45115	-1.4731	0.081712	0.156857	0.763
KEGG_FATT	KEGG_FATT	Details .	42	-0.43256	-1.47033	0.089613	0.156014	0.766
KEGG_INOS	KEGG_INOS	Details .	54	-0.41201	-1.4675	0.100823	0.15453	0.768

KEGG_PERC	KEGG_PERC	Details .	78	-0.42363	-1.45223	0.081395	0.162307	0.784
KEGG_SMAL	KEGG_SMAL	Details .	84	-0.37109	-1.40893	0.100806	0.192833	0.825
KEGG_VASC	KEGG_VASC	Details .	114	-0.34282	-1.40829	0.093886	0.189761	0.826
KEGG_GLYC	KEGG_GLYC	Details .	21	-0.47552	-1.40381	0.109091	0.190172	0.834
KEGG_LEUK	KEGG_LEUK	Details .	116	-0.37536	-1.39925	0.135628	0.190858	0.838
KEGG_INSU	KEGG_INSU	Details .	137	-0.33992	-1.39074	0.070686	0.194001	0.845
KEGG_SYST	KEGG_SYST	Details .	135	-0.41596	-1.35289	0.181308	0.224922	0.889
KEGG_TYRC	KEGG_TYRC	Details .	42	-0.39421	-1.33899	0.141649	0.234515	0.906
KEGG_NITR	KEGG_NITR	Details .	23	-0.41977	-1.33487	0.127745	0.234859	0.912
KEGG_TAST	KEGG_TAST	Details .	51	-0.36602	-1.32744	0.135729	0.238355	0.917
KEGG_NEUR	KEGG_NEUR	Details .	271	-0.29396	-1.32605	0.059794	0.235708	0.917
KEGG_MTOR	KEGG_MTOR	Details .	52	-0.35387	-1.31221	0.1417	0.244644	0.927
KEGG_COLC	KEGG_COLC	Details .	62	-0.35391	-1.30977	0.148515	0.243254	0.929
KEGG_NUCL	KEGG_NUCL	Details .	44	-0.414	-1.30005	0.193613	0.249265	0.942
KEGG_OTHE	KEGG_OTHE	Details .	16	-0.5059	-1.29749	0.193814	0.248107	0.942
KEGG_AMYC	KEGG_AMYC	Details .	53	-0.34926	-1.29688	0.143443	0.245112	0.943
KEGG_CHRC	KEGG_CHRC	Details .	73	-0.34052	-1.29353	0.155419	0.244347	0.944
KEGG_PATH	KEGG_PATH	Details .	325	-0.29735	-1.27641	0.143451	0.256539	0.955
KEGG_NEUR	KEGG_NEUR	Details .	126	-0.32091	-1.27257	0.15121	0.256402	0.957
KEGG_TIGH	KEGG_TIGH	Details .	132	-0.31001	-1.23989	0.193018	0.286685	0.968
KEGG_PANT	KEGG_PANT	Details .	16	-0.4219	-1.23873	0.191257	0.283742	0.968
KEGG_SELE	KEGG_SELE	Details .	26	-0.38521	-1.23313	0.211045	0.285876	0.971
KEGG_GLYC	KEGG_GLYC	Details .	31	-0.36914	-1.21727	0.221757	0.299189	0.979
KEGG_ENDC	KEGG_ENDC	Details .	52	-0.33823	-1.20712	0.234	0.306707	0.983
KEGG_GLYC	KEGG_GLYC	Details .	16	-0.43338	-1.19915	0.260355	0.311424	0.985
KEGG_SPLI	KEGG_SPLI	Details .	127	-0.37389	-1.1979	0.279749	0.308734	0.985
KEGG_PURI	KEGG_PURI	Details .	158	-0.2799	-1.17701	0.250996	0.329139	0.992
KEGG_ALDC	KEGG_ALDC	Details .	42	-0.33739	-1.16654	0.243461	0.336004	0.992
KEGG_LYSI	KEGG_LYSI	Details .	44	-0.34818	-1.16192	0.286869	0.336855	0.992
KEGG_LYSC	KEGG_LYSC	Details .	121	-0.32714	-1.14741	0.307087	0.349937	0.994
KEGG_BLAD	KEGG_BLAD	Details .	42	-0.32394	-1.14695	0.279612	0.345987	0.994
KEGG_THYR	KEGG_THYR	Details .	29	-0.35092	-1.14622	0.280488	0.34274	0.994
KEGG_P53_	KEGG_P53_	Details .	68	-0.31523	-1.14489	0.283096	0.34	0.994
KEGG_CALC	KEGG_CALC	Details .	177	-0.26401	-1.13471	0.262729	0.34728	0.996
KEGG_BASA	KEGG_BASA	Details .	55	-0.30985	-1.13459	0.310757	0.343298	0.996
KEGG_LONG	KEGG_LONG	Details .	70	-0.28542	-1.13309	0.287594	0.341027	0.996
KEGG_GLIC	KEGG_GLIC	Details .	65	-0.28641	-1.08436	0.331984	0.398331	0.998
KEGG_TRYF	KEGG_TRYF	Details .	40	-0.32488	-1.08372	0.359667	0.394738	0.998
KEGG_MELA	KEGG_MELA	Details .	101	-0.27234	-1.08037	0.338086	0.394488	0.998
KEGG_PROG	KEGG_PROG	Details .	85	-0.28549	-1.07709	0.354639	0.394275	0.998
KEGG_ERBE	KEGG_ERBE	Details .	87	-0.27362	-1.07262	0.373984	0.394989	0.998
KEGG_PYRI	KEGG_PYRI	Details .	97	-0.29049	-1.0432	0.398798	0.429759	1
KEGG_PROX	KEGG_PROX	Details .	23	-0.34346	-1.04202	0.42	0.426792	1
KEGG_MATU	KEGG_MATU	Details .	25	-0.35413	-1.03831	0.405941	0.426818	1
KEGG_HOMC	KEGG_HOMC	Details .	28	-0.35387	-1.03603	0.413386	0.425506	1
KEGG_REGU	KEGG_REGU	Details .	35	-0.3335	-1.03382	0.413934	0.424087	1
KEGG_VALI	KEGG_VALI	Details .	44	-0.33763	-1.02202	0.436475	0.435642	1
KEGG_HEDG	KEGG_HEDG	Details .	56	-0.2644	-1.00716	0.421589	0.451471	1
KEGG_HUNT	KEGG_HUNT	Details .	180	-0.28191	-1.00207	0.441118	0.453766	1
KEGG_PRIC	KEGG_PRIC	Details .	35	-0.32563	-0.98906	0.481557	0.466748	1
KEGG_PRIM	KEGG_PRIM	Details .	16	-0.33962	-0.98478	0.469758	0.467767	1

KEGG_BUTAKEGG_BUTANOATE_MET		34	-0.30249	-0.97738	0.458753	0.473104	1
KEGG_GLYCKEGG_GLYCOSAMINOGL		22	-0.33972	-0.9746	0.476483	0.472234	1
KEGG_VASCKEGG_VASOPRESSIN_R		44	-0.28086	-0.96475	0.501018	0.480658	1
KEGG_PHENKEGG_PHENYLALANINE		18	-0.3391	-0.94892	0.512605	0.497607	1
KEGG_RIBCKEGG_RIBOFLAVIN_ME		16	-0.31754	-0.94498	0.526627	0.498102	1
KEGG_GLYCKEGG_GLYCOSYLPHOSP		25	-0.32455	-0.94015	0.510557	0.499967	1
KEGG_ONE_KEGG_ONE_CARBON_PC		17	-0.32659	-0.90305	0.558468	0.548798	1
KEGG_BETAKEGG_BETA_ALANINE_		22	-0.26024	-0.85126	0.656379	0.617154	1
KEGG_PARKKEGG_PARKINSONS_DI		128	-0.27052	-0.82242	0.612774	0.653916	1
KEGG_RIBCKEGG_RIBOSOME		88	-0.35043	-0.78537	0.627409	0.701787	1
KEGG_COMPKEGG_COMPLEMENT_AN		69	-0.27511	-0.70454	0.83871	0.809062	1
KEGG_OLFAKEGG_OLFACTORY_TRA		385	-0.23135	-0.61531	0.99802	0.898843	1

RCA entire set.

RANK AT M	LEADING EDGE
9370	tags=39%, list=17%, signal=46%
6690	tags=30%, list=12%, signal=34%
4094	tags=74%, list=7%, signal=80%
5783	tags=28%, list=10%, signal=31%
4223	tags=36%, list=7%, signal=39%
6675	tags=65%, list=12%, signal=74%
8327	tags=33%, list=15%, signal=39%
3848	tags=50%, list=7%, signal=54%
3338	tags=24%, list=6%, signal=26%
3354	tags=54%, list=6%, signal=57%
6334	tags=44%, list=11%, signal=49%
3848	tags=59%, list=7%, signal=63%
5212	tags=41%, list=9%, signal=45%
3848	tags=74%, list=7%, signal=80%
9025	tags=33%, list=16%, signal=39%
6757	tags=47%, list=12%, signal=53%
5783	tags=32%, list=10%, signal=35%
3522	tags=20%, list=6%, signal=21%
7962	tags=76%, list=14%, signal=88%
9852	tags=38%, list=17%, signal=46%
4062	tags=25%, list=7%, signal=27%
8755	tags=29%, list=15%, signal=34%
10816	tags=38%, list=19%, signal=47%
7785	tags=36%, list=14%, signal=42%
7962	tags=35%, list=14%, signal=40%
9370	tags=32%, list=17%, signal=39%
6486	tags=43%, list=11%, signal=48%
9913	tags=34%, list=18%, signal=41%
8154	tags=32%, list=14%, signal=38%
6918	tags=37%, list=12%, signal=42%
6819	tags=22%, list=12%, signal=25%
5783	tags=21%, list=10%, signal=23%
4112	tags=31%, list=7%, signal=33%
8155	tags=40%, list=14%, signal=47%
14028	tags=34%, list=25%, signal=45%
6485	tags=22%, list=11%, signal=25%
3731	tags=22%, list=7%, signal=24%
9378	tags=41%, list=17%, signal=49%
11970	tags=37%, list=21%, signal=47%
9370	tags=32%, list=17%, signal=38%
6461	tags=23%, list=11%, signal=26%
7459	tags=21%, list=13%, signal=25%
6632	tags=29%, list=12%, signal=33%
2444	tags=17%, list=4%, signal=17%
8125	tags=22%, list=14%, signal=26%
14077	tags=40%, list=25%, signal=54%
6788	tags=29%, list=12%, signal=33%
10053	tags=29%, list=18%, signal=35%
9913	tags=33%, list=18%, signal=40%

6555 tags=23%, list=12%, signal=26%
6128 tags=24%, list=11%, signal=27%
6740 tags=20%, list=12%, signal=23%
3390 tags=19%, list=6%, signal=20%
2203 tags=16%, list=4%, signal=16%
9259 tags=26%, list=16%, signal=30%
3354 tags=16%, list=6%, signal=16%
7105 tags=21%, list=13%, signal=24%
8405 tags=22%, list=15%, signal=26%
19400 tags=41%, list=34%, signal=63%
10918 tags=20%, list=19%, signal=25%
7828 tags=23%, list=14%, signal=27%
7828 tags=27%, list=14%, signal=32%
12733 tags=36%, list=23%, signal=47%
6604 tags=31%, list=12%, signal=35%
7577 tags=25%, list=13%, signal=28%
4515 tags=18%, list=8%, signal=19%
6356 tags=18%, list=11%, signal=21%
9537 tags=27%, list=17%, signal=32%
8984 tags=22%, list=16%, signal=26%
15417 tags=44%, list=27%, signal=60%
4694 tags=15%, list=8%, signal=17%
5621 tags=16%, list=10%, signal=18%
7828 tags=25%, list=14%, signal=29%
10365 tags=31%, list=18%, signal=38%
6853 tags=20%, list=12%, signal=23%
10312 tags=25%, list=18%, signal=30%
8128 tags=21%, list=14%, signal=25%
6677 tags=23%, list=12%, signal=26%
8272 tags=25%, list=15%, signal=29%
6356 tags=21%, list=11%, signal=24%
5388 tags=24%, list=10%, signal=27%
8302 tags=24%, list=15%, signal=28%
10823 tags=23%, list=19%, signal=28%
7111 tags=20%, list=13%, signal=23%
7838 tags=17%, list=14%, signal=20%
9259 tags=18%, list=16%, signal=22%
8872 tags=25%, list=16%, signal=30%
4288 tags=14%, list=8%, signal=15%
8120 tags=21%, list=14%, signal=25%
3645 tags=13%, list=6%, signal=13%
12733 tags=32%, list=23%, signal=41%
8559 tags=22%, list=15%, signal=26%
9652 tags=20%, list=17%, signal=24%
8498 tags=21%, list=15%, signal=25%
14310 tags=29%, list=25%, signal=38%
12599 tags=32%, list=22%, signal=41%
6263 tags=16%, list=11%, signal=18%
11539 tags=25%, list=20%, signal=31%
5908 tags=20%, list=10%, signal=22%
11879 tags=25%, list=21%, signal=32%

```
 7534 tags=21%,  list=13%,  signal=24%
 5157 tags=23%,  list=9%,  signal=25%
 3522 tags=11%,  list=6%,  signal=12%
 9684 tags=22%,  list=17%,  signal=27%
 9269 tags=25%,  list=16%,  signal=30%
13664 tags=32%,  list=24%,  signal=42%
13421 tags=35%,  list=24%,  signal=46%
13658 tags=32%,  list=24%,  signal=42%
11574 tags=24%,  list=20%,  signal=30%
14757 tags=40%,  list=26%,  signal=54%
 9682 tags=22%,  list=17%,  signal=26%
34750 tags=70%,  list=61%,  signal=180%
```
