# Supplementary material for: A Novel Prognostic Ferroptosis-Related lncRNA Signature Associated with Immune Landscape in Invasive Breast Cancer
Source: Dis Markers. 2022 Mar 20;2022:9168556. doi: 10.1155/2022/9168556 (PMC8961446; doi:10.1155/2022/9168556)
Supplement: Supplementary 3 — Figure S3: gene enrichment analysis for the ferroptosis-related lncRNAs of the low-risk group based on the TCGA-BRCA entire set. [file 9168556.f3.pdf]

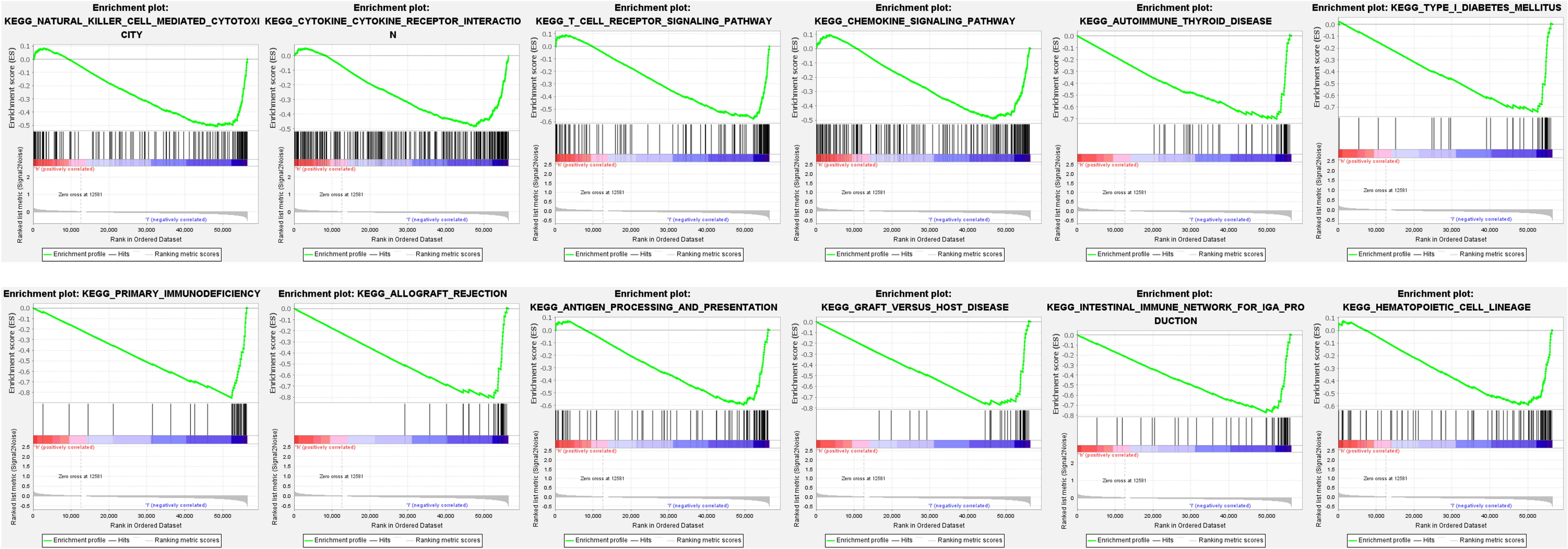
Enrichment plot: KEGG_NATURAL_KILLER_CELL_MEDIATED_CYTOTOXICITY
Enrichment plot: KEGG_CYTOKINE_CYTOKINE_RECEPTOR_INTERACTION
Enrichment plot: KEGG_T_CELL_RECEPTOR_SIGNALING_PATHWAY
Enrichment plot: KEGG_CHEMOKINE_SIGNALING_PATHWAY
Enrichment plot: KEGG_AUTOIMMUNE_THYROID_DISEASE
Enrichment plot: KEGG_TYPE_I_DIABETES_MELLITUS
Enrichment plot: KEGG_PRIMARY_IMMUNODEFICIENCY
Enrichment plot: KEGG_ALLOGRAFT_REJECTION
Enrichment plot: KEGG_ANTIGEN_PROCESSING_AND_PRESENTATION
Enrichment plot: KEGG_GRAFT_VERSUS_HOST_DISEASE
Enrichment plot: KEGG_INTESTINAL_IMMUNE_NETWORK_FOR_IGA_PRODUCTION
Enrichment plot: KEGG_HEMATOPOIETIC_CELL_LINEAGE
Enrichment score (ES)
Ranked list metric (Signal2Noise)
'h' (positively correlated)
'l' (negatively correlated)
Zero cross at 12581
Rank in Ordered Dataset
Enrichment profile
Hits
Ranking metric scores
